# Aqueous Inks of Pristine Graphene for 3D Printed Microsupercapacitors with High Capacitance

**DOI:** 10.1021/acsnano.1c06535

**Published:** 2021-09-07

**Authors:** Stefano Tagliaferri, Goli Nagaraju, Apostolos Panagiotopoulos, Mauro Och, Gang Cheng, Francesco Iacoviello, Cecilia Mattevi

**Affiliations:** † Department of Materials, Imperial College London, London SW7 2AZ, United Kingdom; ‡ Electrochemical Innovation Lab, Department of Chemical Engineering, University College London, London WC1E 7JE, U.K.

**Keywords:** 3D printing, pristine graphene, conductivity, capacitance, printed microsupercapacitors

## Abstract

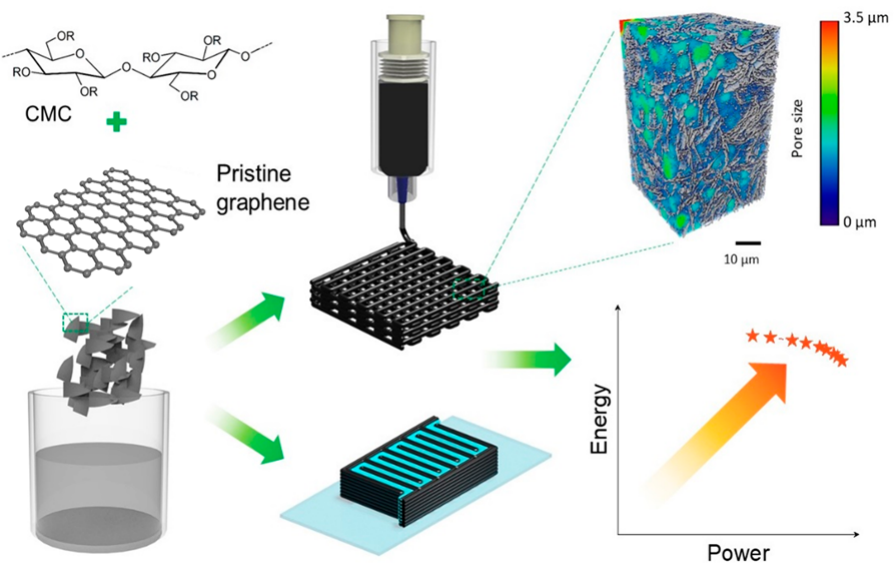

Three-dimensional (3D) printing is gaining importance as a sustainable route for the fabrication of high-performance energy storage devices. It enables the streamlined manufacture of devices with programmable geometry at different length scales down to micron-sized dimensions. Miniaturized energy storage devices are fundamental components for on-chip technologies to enable energy autonomy. In this work, we demonstrate 3D printed microsupercapacitor electrodes from aqueous inks of pristine graphene without the need of high temperature processing and functional additives. With an intrinsic electrical conductivity of ∼1370 S m^–1^ and rationally designed architectures, the symmetric microsupercapacitors exhibit an exceptional areal capacitance of 1.57 F cm^–2^ at 2 mA cm^–2^ which is retained over 72% after repeated voltage holding tests. The areal power density (0.968 mW cm^–2^) and areal energy density (51.2 μWh cm^–2^) outperform the ones of previously reported carbon-based supercapacitors which have been either 3D or inkjet printed. Moreover, a current collector-free interdigitated microsupercapacitor combined with a gel electrolyte provides electrochemical performance approaching the one of devices with liquid-like ion transport properties. Our studies provide a sustainable and low-cost approach to fabricate efficient energy storage devices with programmable geometry.

The growing demand for eco-benign and high-performance energy storage (ES) devices with integrated portable designs for consumer electronics and, more recently, on-chip technologies has sparked significant progress in the field.^1−3^ More specifically, supercapacitors have been attracting increasing attention owing to their high power density, rapid charge–discharge rate, excellent cycling durability, and safety.^4^ Carbon-based materials have long been the chief choice for the fabrication of low-cost and efficient supercapacitors;^4^ among those, graphene^5−8^ has demonstrated superior performance compared to other carbon materials.^9^ It presents high intrinsic capacitance, high mechanical strength and flexibility, and excellent electrical conductivity. This combination of properties allows the formation of 3D percolating structures which act as fast pathways for electron transport.^10^ Porous 3D graphene electrodes are able to provide high-energy density owing to the reduced transport resistance and accessible surface area to charge storage processes.^11−13^ In particular, 3D printed electrodes over small footprint areas with customized porous architectures are considered as the state-of-the-art configuration for high-performance graphene microsupercapacitors.^14,15^ 3D printed graphene microsupercapacitors, presenting footprint areas ∼1 cm^2^ and submillimeter-scale features, can be used as the energy storage component in autonomous electronic devices.^3^ Compared to rigid/dense film-like materials, 3D printed porous graphene microelectrodes offer higher electrochemical surface area. Having high surface area and porosity, the printed electrodes facilitate the rapid penetration of the electrolyte into the active material, increasing capacitance and rate performance, respectively. Additionally, 3D printed porous micron-sized electrodes provide shorter ion diffusion paths which increase the rate capability at higher currents.^16^ Among 3D printing techniques, extrusion-based 3D printing can provide great structural control on the manufacturing of electrodes for energy devices, allowing the tailored fabrication of macropores and uninterrupted pathways which promote electrolyte infiltration and charge transport.^16−18^ Although graphene has been widely used as a functional additive in Fused Deposition Modeling, this 3D printing process requires high amounts of thermoplastic polymers which lack electroactive properties.^19,20^ Extrusion-based 3D printing techniques which do not rely on molten polymers to fabricate graphene structures are therefore preferred, such as Direct Ink Writing (DIW), as it is the case of this work. More specifically, we have used a robocasting printing technique which relies on a gantry robot for spanning the deposition nozzle to fabricate structures *via* the layer-by-layer continuous extrusion of a viscoplastic ink.

The formulation of printable inks for DIW with high loading of graphene is usually a challenge, especially when water-based formulations are considered. The hydrophobic character of pristine graphene makes it unstable in aqueous dispersions, hindering processability.^21,22^ Consequently, the DIW of graphene electrodes has been dominated by the use of graphene oxide (GO) and reduced graphene oxide (rGO) in place of pristine graphene (PG). GO can be easily dispersed in water at very high concentrations (∼10–100 mg/mL) owing to its hydrophilic moieties.^23,24^ The hydroxyl and carboxylic groups on GO promote the formation of hydrogen bonds between flakes, resulting in gel-like rheological properties which are necessary to achieve good shape retention during DIW.^25,26^ However, GO is an electrical insulator,^27,28^ and after printing, GO electrodes require reduction processes which involve high temperatures (>1000 °C)^16,29−31^ and hazardous chemicals to eliminate the oxygen functional groups and impart some electrical conductivity.^32^ Nonetheless, it is known that GO remains highly defective even after thermal or chemical reduction, presenting residual oxygen moieties and topological defects in the sp^2^ carbon networks which diminish the electronic transport.^10,33,34^ As high conductivity is essential in 3D printed microsupercapacitors to limit internal losses and preserve good performance at high rates, highly conductive additives are normally incorporated in the GO inks, increasing the complexity, processing, and cost of the inks.^31,35^ Recently, a novel class of inks for extrusion processes has emerged, called capillary inks, which present the advantage of eliminating or reducing the postprocessing of printed structures.^36−38^ They are based on the formation of a network of capillary bridges between particles, enabled by a secondary liquid which is chosen to match the surface tension of the particles.^39^ The bridges act as reversible links, which are able to deform and break during an extrusion process and to gradually reform once the material is at rest, providing a yield stress and shear-thinning rheology.^40^ This strategy has been applied to graphene nanoplatelets allowing the formulation of aqueous inks from pristine graphene without the need of GO.^36^

Herein, we demonstrate high-performance and customized 3D printed symmetric microsupercapacitors based on highly concentrated pristine graphene (PG) aqueous inks. The inks present suitable rheological characteristics for printing mechanically robust structures of several layers; additionally, the printed structures do not require postprinting processes involving hazardous chemicals and high temperatures. Structural and electrical characterization revealed that the printed PG electrodes present high porosity (average pore size ∼1.4 μm) and excellent electrical conductivity. Benefiting from the synergistic combination of structural features, 3D printed porous PG electrodes provide pathways for rapid ion diffusion and fast charge transport during the energy storage process. In a symmetric supercapacitor configuration, the 3D printed PG electrodes demonstrate excellent areal capacitance of ∼1.57 F cm^–2^ at 2 mA cm^–2^, areal energy density of 51.2 μWh cm^–2^, and areal power density of 0.968 mW cm^–2^ which are prime within the state-of-the-art of printed carbon- and graphene-based supercapacitors. Moreover, we demonstrate prepatterned and current collector-free quasi-solid-state interdigitated microsupercapacitors with PG platelet electrodes in a water-based gel electrolyte. The demonstrated 3D printed PG electrodes are expected to promote the development of sustainable and cost-effective energy storage devices with high performance.

## Results and Discussion

**Figure 1:**
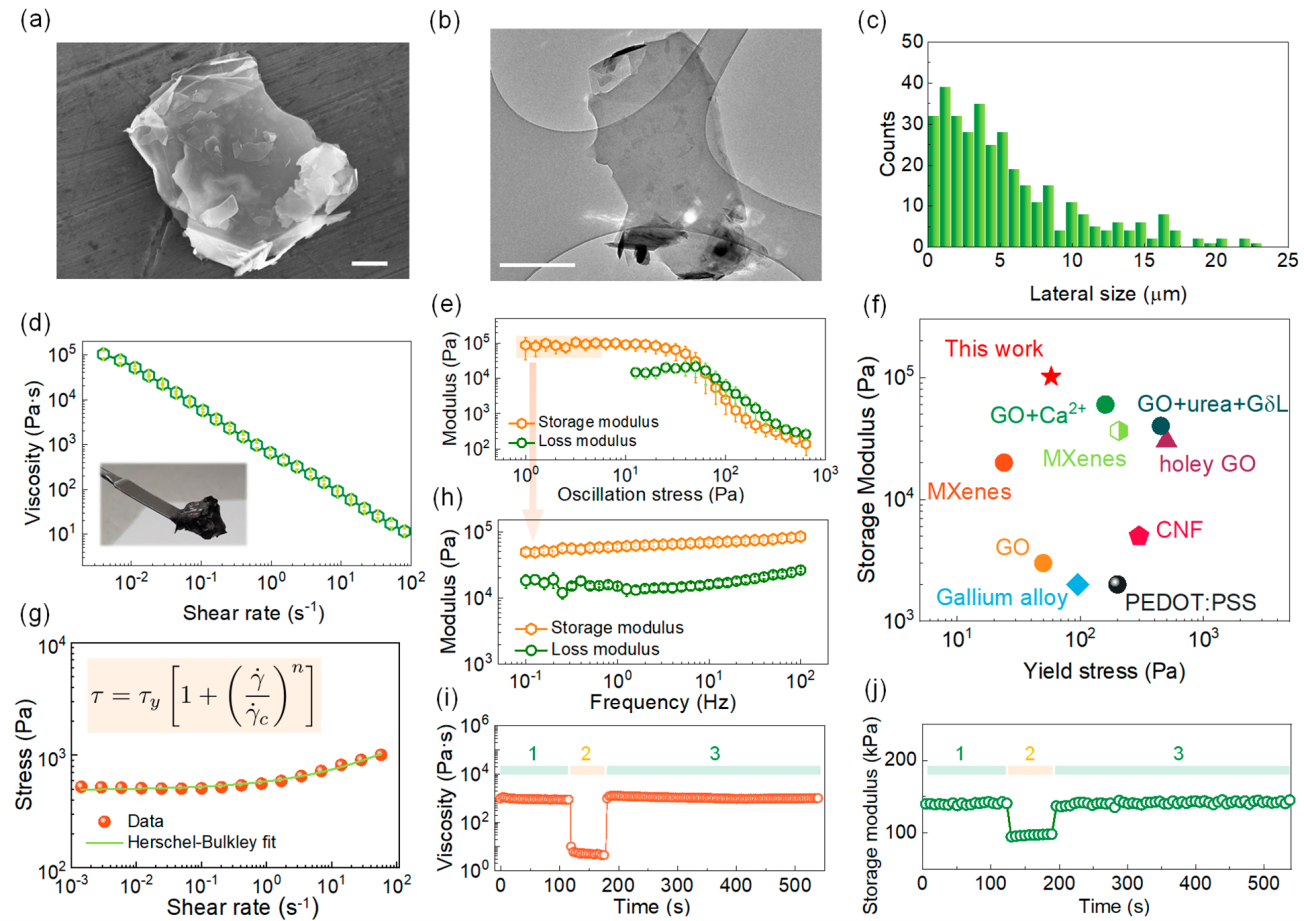
a,b) SEM and TEM images of the two-dimensional PG platelets used in this work to formulate the graphene inks (scale bars 900 nm and 1 μm, respectively). c) Histogram reporting the lateral size of PG platelets, as determined from SEM imaging. d) Viscosity curve for the PG inks and (inset) picture of the PG ink after homogenization and e) amplitude sweep tests showing the storage (*G*′) and loss (*G*″) moduli as a function of oscillatory stress. f) Comparative yield stress and storage modulus of the PG ink with previous literature based on DIW inks for an energy device (references can be found in Table S2). g) Shear stress curve for the PG ink and Herschel-Bulkley equation used to fit the corresponding curve (inset). h) Frequency sweep test performed in the linear viscoelastic region for the PG inks and j) three interval thixotropy tests (3ITTs) showing the recovery of viscosity (i) and storage modulus (j) after a high-shear step (step 2, highlighted in orange).

The PG inks for Direct Ink Writing were formulated using commercial 2D-architectured PG platelets with an average thickness of 6–8 nm. The graphene platelets present an average lateral size of ∼6 μm as shown by the SEM and TEM images in Figures 1a–c and S1. Raman spectroscopy analysis revealed a thickness of the PG platelets of more than 6 layers (Figures S1 and S2), confirmed by AFM characterization which indicated that the platelets consist of ∼10 layers (∼3.5 nm, Figure S1c,d). The DIW inks were formulated by dispersing the PG platelets in water with a small amount of cellulose viscosifier (∼4 wt %) and 1-octanol as the secondary liquid (∼2% with respect to water).^36,37^ After high-speed planetary mixing, a homogeneous slurry was obtained that could be readily used as ink for 3D printing (Figure S3a,b). The printability of the slurry was investigated through rheological analysis using a rotational rheometer to demonstrate the feasibility of the PG ink for the fabrication of supercapacitor electrodes. Roughened plates were employed during the shear tests to avoid wall slip and other experimental artifacts which would reduce the reliability of the results (Figure S3c). The shear curve in Figure 1d presents a significant drop in viscosity over the range of shear rates investigated, signifying a prominent shear-thinning behavior which facilitates the extrusion of the ink through fine nozzles during the printing process, allowing the fabrication of electrodes with miniaturized features.^17,41^ In particular, the shear thinning of the PG ink is caused by the progressive disruption of the capillary bridges between the PG particles at increasing shear rates, as already observed for similar suspensions.^40^ The stress curve (Figure 1g) shows a plateau in the low-rate region, suggesting the existence of a finite yield stress below which the ink is unable to flow. The yield stress and shear-thinning rheology of the PG inks could be fitted with the Herschel-Bulkley equation (inset in Figure 1g), which is an empirical model widely used in the description of DIW inks.^42^ A flow index *n* < 1 was found from the fitting of the experimental data, which is indicative of the high shear-thinning response (data reported in Table S1).

The existence of a yield stress is a fundamental feature in DIW inks, which allows the fabrication of self-standing electrodes at room temperature. The yielding behavior was further investigated through oscillatory rheometry (Figure 1e). At low oscillatory stress, the viscoelastic response of the material is dominated by the elastic component (tan δ ∼ 0.2, Figure S4b) with a very high storage modulus of ∼10^5^ Pa. In this region (known as the linear viscoelastic region, LVE), the storage modulus is almost independent of the oscillation frequency (Figure 1h), a feature that is common in a gelled system presenting a space-spanning network of bonds. When a stress over ∼60 Pa is applied to the PG inks, the viscous response becomes predominant (*G*″ > *G*′) indicating the destruction of the capillary network and the onset of the flow. A comparison between the storage modulus and yield stress, *i.e*., the two main parameters used to describe printability, of our PG inks with other DIW inks for energy devices is reported in Figure 1f. Notably, the PG inks exhibit a very high storage modulus when compared to MXenes and GO, which prevents shape deformation, such as sagging and buckling under the effect of gravity.^17,43^

The recovery of the structural properties of the PG ink after shear was investigated through three interval thixotropy tests (3ITTs). A fast and complete structural restoration after ejection from the printing nozzle is necessary to obtain the desired electrode geometry and to avoid uncontrolled spread and collapse of the ink filament. This is especially important in printed energy devices, where the electrode geometry influences the transport of ions and electrons and ultimately the performance of the device. Both the viscosity and storage modulus of the PG inks were taken into account to evaluate the restructuring kinetics (Figure 1i,j). To perform the viscosity recovery tests (Figure 1i), the ink was first sheared at a low rate (0.1 s^–1^ for 120 s, step 1) to equilibrate its microstructure, then at a high rate (100 s^–1^ for 60 s, step 2) to further break the capillary network, and finally at a low rate of 0.1 s^–1^ for 120 s (step 3) to monitor the viscosity rebuild. Similarly, in the storage modulus recovery tests (Figure 1j), an initial low-stress oscillatory step (10 Pa, step 1) was employed to evaluate the rest modulus, followed by a high-stress step (100 Pa, step 2) to destroy the inner structure of the sample and a final low-stress step (10 Pa, step 3) to evaluate the restructuring time. The stress value during step 2 (100 Pa, leading to a strain ∼0.1%) was chosen to be higher than the yield stress (∼60 Pa), so to ensure the destruction of the ink microstructure. An almost instantaneous recovery of both the viscosity and the storage modulus occurs after step 2 (Figure 1i,j), suggesting a fast restructuring of the PG ink. When much larger strain values were employed in step 2, the recovery of the storage modulus was not complete (Figure S4c). Nonetheless, the storage modulus was able to quickly recover to values >10^4^ Pa which are still in the acceptable range for DIW deposition, as shown in Figure 1f. The high storage modulus and fast structural recovery of the PG inks allowed to obtain electrodes with tailored architectures, presenting no shape deformation after deposition and drying which might affect the transport of electrons and penetration of the electrolyte into the printed structures.

**Figure 2:**
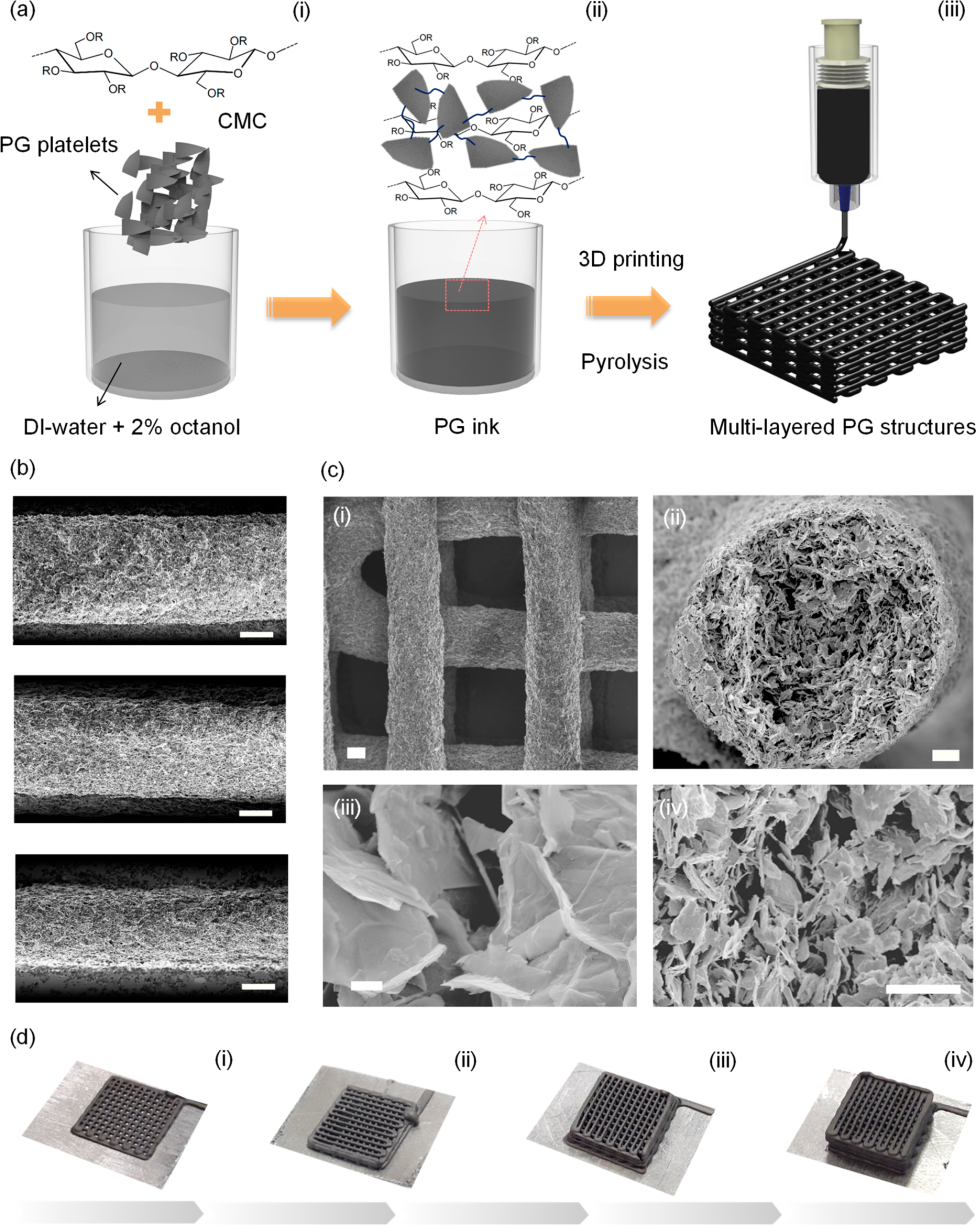
a) Schematic showing the formulation and printing of the PG ink, (i) PG platelets are mixed with an aqueous solution of carboxymethyl cellulose (CMC) to which 1-octanol is added to form intersheet bridges; the dispersion is homogenized (ii) and 3D printed (iii) to fabricate woodpile and interdigitated electrodes. b) SEM images of printed PG lines obtained using nozzles of different diameters: 410, 250, and 200 μm (top to bottom, respectively), scale bars are 100 μm. c) SEM images of a woodpile electrode (i) and cross-section images of a printed strut at increasing magnification (ii)–(iv), scale bars are 100, 30, 20, and 1 μm, respectively. d) Pictures of woodpile electrodes (1 cm^2^ footprint area) presenting an increasing number of layers: 2 (i), 4 (ii), 6 (iii), and 8 (iv) layers.

The PG ink was used to fabricate customized woodpile electrodes with multiple layers for microsupercapacitors *via* DIW, as outlined in Figure 2a. The inks could be smoothly extruded through nozzles as fine as 200 μm (Figures 2b and S5a), providing high resolution in the deposition process. The printed struts also presented negligible shrinkage during solvent evaporation and subsequent annealing at 350 °C. The woodpile electrodes could be deposited on a variety of different substrates, including graphite paper, glass, ITO, copper, and Si/SiO_2_ (Figure S5b). The woodpile structure (Figure 2c(i)) exhibits shape retention after printing and solvent evaporation, with parallel cylindrical rods made of an interconnected network of PG platelets, as demonstrated by the cross-section SEM (Figures 2c(ii)–(iv) and S5d). Additionally, the porous features and cavities inside the printed PG electrode (Figure 2c(iii)) enable rapid diffusion of the electrolyte ions into the interior of the printed electrode. The geometric printability, calculated according to eq S1 and Figure S3d, is almost 1 (∼0.996), demonstrating optimal shape preservation and the absence of morphological defects in the electrode design. The electrode thickness and the areal density of active material can be easily controlled during the printing process by progressively increasing the number of stacked layers deposited by the nozzle. Woodpile electrodes ranging from 2 to 8 layers (∼8.9 mg to ∼33 mg) were fabricated, as shown in Figures 2d and S5c.

**Figure 3:**
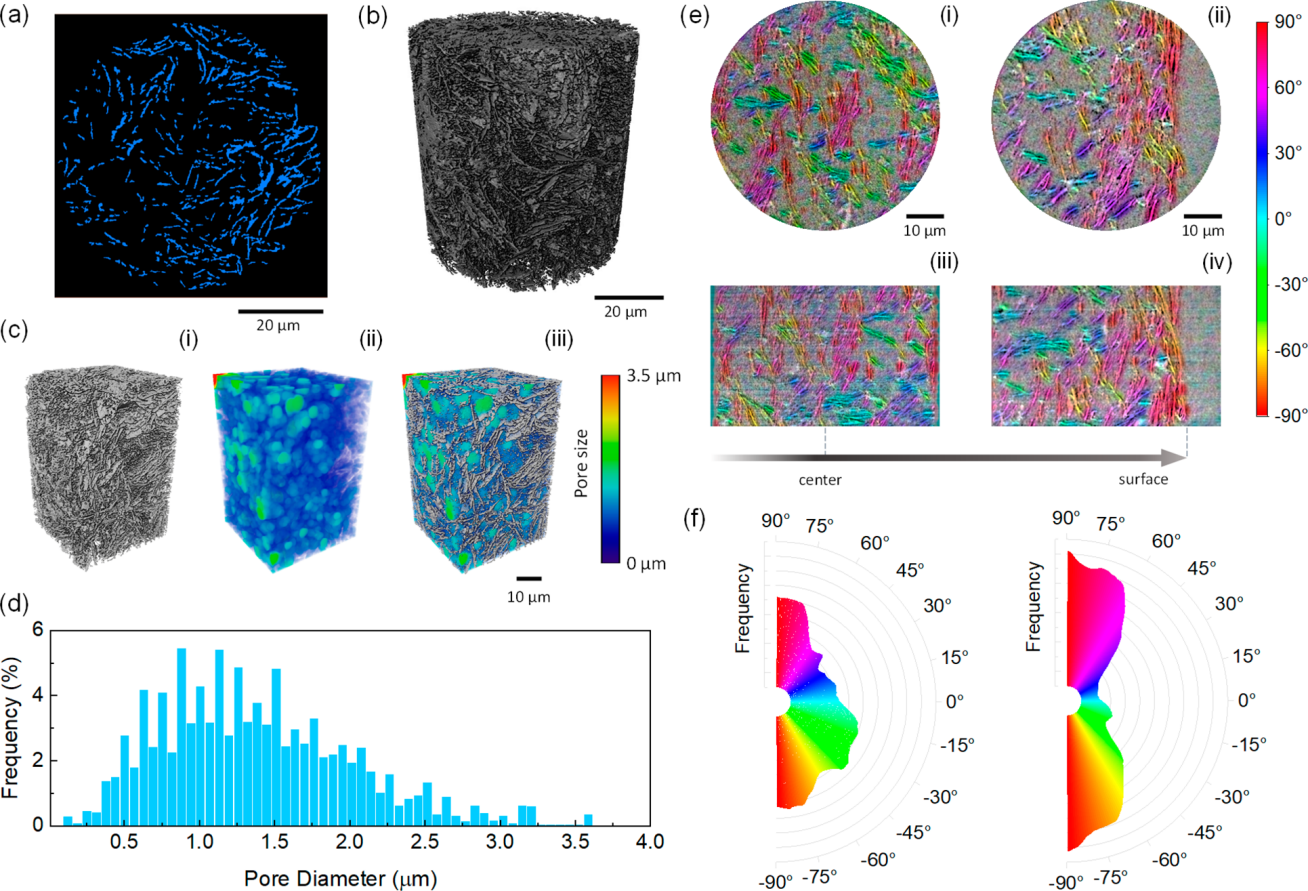
a,b) Reconstruction of a printed filament obtained from X-ray Nano-Computed Tomography: (a) slice perpendicular to the filament axis and (b) 3D rendering of the central region of the filament. c) 3D visualization of the region considered to calculate porosity: (i) PG platelets, (ii) pores, and (iii) simultaneous visualization of PG platelets and pores. d) Pore size distribution in the printed filament. e) Orientation of the platelets in the central (i), (iii) and outer (ii), (iv) regions of the filament: (i) and (ii) represent slices perpendicular to the filament axis, while (iii) and (iv) represent slices along the filament axis. f) Radial histograms presenting the orientation of platelets in the central (left) and outer (right) regions of the printed filament, respectively.

The porosity of the printed electrodes and the local disposition of platelets inside the extruded filaments contribute in determining the electronic and ionic transport, hence the performance of the supercapacitors.^29,44^ X-ray Nano-Computed Tomography was used to obtain detailed microstructural information by mapping and reconstructing selected regions of the printed filaments. The reconstructed 3D model presented in Figure 3a,b confirms the existence of an uninterrupted network of platelets across the area sampled, that can act as a continuous pathway for the transport of electrons to the current collector. The presence of abundant pores with an average diameter of ∼1.4 μm between the platelets (Figure 3c,d) is beneficial to the electrolyte infiltration and ion diffusion. The tomographic scan reported in Figure 3a indicates that the PG platelets tend to align parallel to the surface in the outer region of the filament, whereas they show a random orientation in the inner core, probably owing to the different flow experienced by the two regions during printing.^45,46^ This phenomenon can be explained as the ink is subjected to both shear and elongational forces during the extrusion process that tend to align the anisotropic particles along the flow direction.^47^ In yield stress fluids, the orientation of particles occurs when the applied stress is higher than the yield stress of the ink.^47,48^ In our ink, this occurs in the exterior region of the ink-filament, which is able to flow, whereas the central region remains unyielded, presenting a solid-like plug flow with no reorientation of the particles. The radius of the unyielded region depends on the shear stress imposed on the material, becoming negligible when the maximum shear stress is significantly larger than the yield stress.^49^ The platelet orientation angle was quantified calculating the gradient structure tensor of the tomographic images (via the OrientationJ plug-in for ImageJ) in the central and outer region of the filament, respectively. The orientation maps presented in Figure 3e indicate that the platelets tend to align parallel to the surface in a region ∼15 μm from the surface, whereas they are randomly oriented in the inner core, across ∼380 μm in diameter, which corresponds to the unyielded region of the ink. It is worth noting that this inner region is significantly larger than the one expected for a Herschel-Bulkley ink flowing in a cylindrical nozzle (∼140 μm, as detailed in the SI, eqs S2 and S3), possibly because the ink has a short residence time (∼1 s) inside the nozzle. In fact, the PG platelets require a finite time to completely align along the flow direction, and the alignment time scales as the inverse of the shear rate.^47^ Only the platelets closest to the nozzle walls, which experience the highest shear rate, can reorient in the limited time that the ink spends inside the nozzle, leading to a region of aligned particles. The presence of an outer shell of highly oriented platelets could enhance electronic transport with respect to a randomly oriented microstructure.^50^

**Figure 4:**
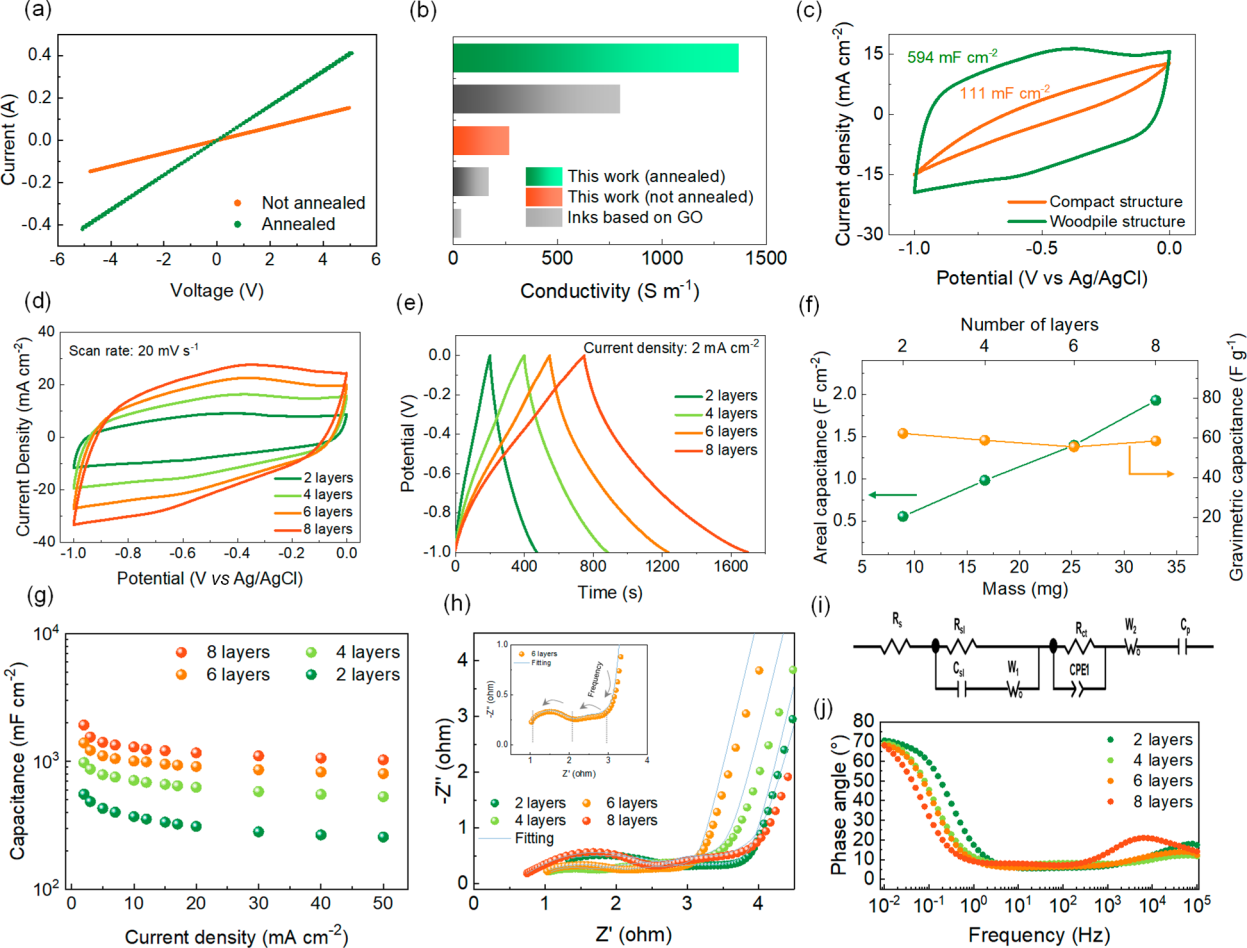
a) I–V curve for the PG ink before and after the annealing process. b) Electrical conductivity of the PG ink before and after the annealing process compared with three different DIW inks based on GO (references were included in Table S3). c–i) Electrochemical performance of printed electrodes in a three-electrode system. c) CV curves for a 4-layer woodpile (green) and compact (orange) PG structures, d,e) CV and GCD curves for woodpile electrodes at an increasing number of layers, and f) comparison of the areal and gravimetric capacitance of the woodpile electrodes with a different number of layers. g) Areal capacitance of PG electrodes (2 to 8 layers) at different current densities, h) EIS curves on 2- to 8-layer electrodes between 0.01 Hz and 100 kHz, (i) EIS fitting circuit, and j) Bode plots for the woodpile electrodes with different numbers of layers.

The conductivity of the PG ink was evaluated through four-electrode measurements (Figures 4a and S6 and eq S4). After drying in air, the inks exhibited an electrical conductivity as high as ∼270 S m^–1^, which is already greater than the conductivity of GO inks after complete reduction (Figure 4b).^18,29^ The electrical properties of the PG ink could be further improved with a mild annealing at 350 °C, which increased the conductivity further to ∼1370 S m^–1^ through the removal of the printing additives. The low-temperature annealing required by the PG structures is significantly more energy-efficient, fast, and sustainable than the thermal reduction of GO electrodes, resulting in a nearly one-step manufacturing process for the printed microsupercapacitors. Additionally, the high electrical conductivity of the printed electrodes can ensure fast electrode kinetics and reduced internal impedance, promoting good rate performance during electrochemical processes.^51^ The feasibility of the woodpile electrodes for the fabrication of microsupercapacitors was demonstrated *via* three-electrode tests in 1 M LiOH at room temperature. Among the electrolytes considered, 1 M LiOH enabled the highest capacitance values, as demonstrated by the cyclic voltammetry tests in Figure S7a,b. To investigate the influence of the electrode geometry and thickness on the electrochemical performance, woodpile electrodes consisting of 2 to 8 layers and a 4-layer compact electrode were fabricated. Although three-dimensional compact electrodes present higher volumetric density of active material, the actual portion of the active material which can be used for charge storage is usually small, owing to the reduced accessible surface area which ultimately determines the electrochemical surface area. The cyclic voltammetry (CV) curves of 4-layer compact and woodpile electrodes are compared in Figure 4c. Despite its large mass (58.3 mg), the compact electrode provides a small areal capacitance of 111 mF cm^–2^ at a scan rate of 20 mV s^–1^ and poor electrochemical performance (Figure S15). In contrast, the woodpile electrode, with its regular macrochannels and accessible surface (Figure S7c,e), can be better infiltrated by the electrolyte, resulting in a capacitance of 594 mF cm^–2^ at a mass loading of only 16.7 mg cm^–2^. The CV of the 2-layer electrode presented in Figure 4d exhibits a quasi-rectangular shape in a potential window of 1 V, which is preserved when increasing the number of layers up to 8, indicating the capacitive nature of the charge storage process. Both the area of the CV curves and the discharge time in galvanostatic charge–discharge tests (Figure 4e) increase linearly with the number of layers, resulting in a direct proportionality between the electrode mass and areal capacitance (Figure 4f). In contrast, the gravimetric capacitance remains approximately constant with increasing mass loading of the electrode, indicating that ionic transport is not significantly hindered in thicker structures. The specific performance of the 3D printed electrodes can therefore be efficiently tuned by adjusting the mass of printed material. The rate performance of 3D electrodes with different thicknesses is presented in Figures 4g, S7d, S8, and S9. Notably, the fraction of the initial capacitance which is preserved at high current densities (50 mA cm^–2^) increases with the electrode mass up to 6 layers, owing to the optimized geometry and high electrical conductivity of the electrodes, which promote fast charge transport in several-millimeter thick structures. The kinetics of charge transport in the PG electrodes with different layer numbers was further investigated using electrochemical impedance spectroscopy (EIS). The Nyquist plot for the 2-, 4-, 6-, and 8-layer PG electrodes is shown in Figure 4h, with the magnified portion of the high-frequency region for the 6-layer electrode displayed in the inset. In particular, the high-frequency region of the EIS curves describes the interfacial charge transfer resistance, whereas the low-frequency response can be attributed to the capacitive behavior of the electrodes. At high frequency, the intercept at the real axis *Z*′ represents the bulk solution resistance, which is the combination of the ionic resistance of the electrolyte, the intrinsic resistance of the electroactive materials, and the contact resistance at the interfaces between the electroactive material and the electrode. The reported Nyquist plots were modeled with ZView using the equivalent circuit shown in Figure 4i. The fitted equivalent circuit presents an ohmic resistor (*R*_s_) representing the combination of the ionic resistance of the electrolyte, the intrinsic resistance of the current collector, and the material/substrate interface resistance; whereas the parallel connections *R*_sl_ and *C*_sl_ represent the charge transfer in the surface layer of the electrode material. The diffusion transport can be described by introducing a Warburg resistance (*W*_1_). The faradaic charge transfer resistance across the electrode/electrolyte interface is referred to as *R*_ct_, and the double layer capacitance (*C*_dl_) was connected parallel with *R*_ct_. At extreme low frequencies, the series connection of a Warburg resistance (*W*_2_) and a capacitance (*C*_p_) was included, which represents the diffusion of the electrolyte and the pseudocapacitance, respectively. The resulting fitted parameters of the EIS plots are presented in Table S6. The 6-layer PG electrode results in possessing lower charge transfer resistance and high capacitive behavior from the electroactive material compared to the other electrodes. It is believed that the higher surface area and macro/microporous channels of PG electrodes can significantly raise the ionic mobility of the electrolyte and reduce the solution resistance. The resultant Bode plots for PG electrodes for different layer numbers are shown in Figure 4j. At lower frequencies, the phase angle approaches −90° indicating that the supercapacitor electrodes behave almost like an ideal capacitor. The Bode plot further suggests that the 6-layer PG electrode possesses high capacitive behavior with a low-frequency phase angle of ∼−68.3°. Even though the Bode phase angle is very close for the 2- and 4-layer electrodes, the low overall resistance of the 6-layer PG electrode makes it more interesting with respect to the 4-layer electrodes. Considering the excellent electrochemical performance, including high-rate capability, low charge transfer resistance, and ideal capacitive nature,^5^ the 6-layer PG electrode could be expected to demonstrate high-energy storage properties in symmetric supercapacitors.

**Figure 5:**
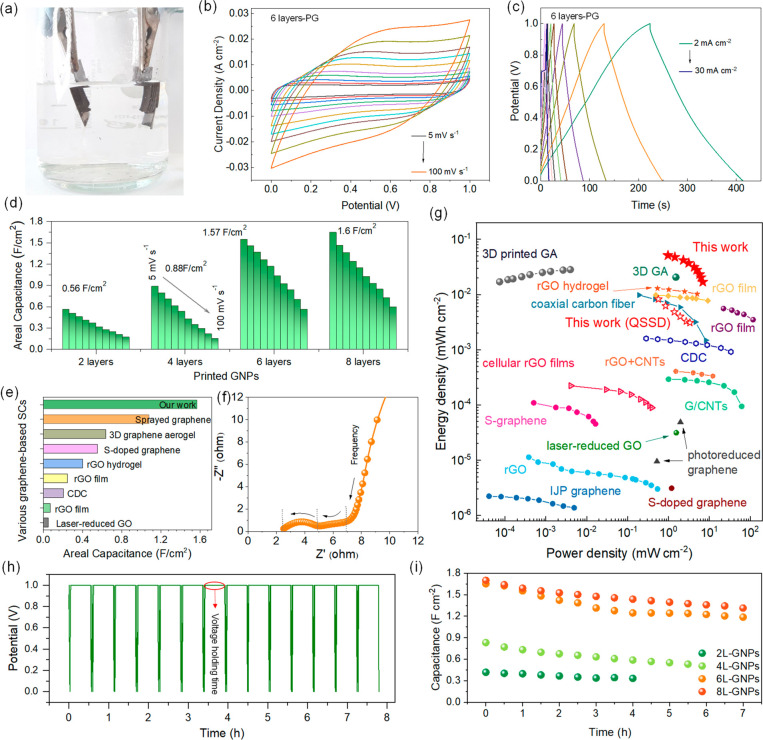
a) Picture of the testing system for the PG woodpile electrodes in a symmetric two-electrode configuration. b) CV curves for a 6-layer symmetric device at different scan rates (5 to 100 mV s^–1^) and c) GCD for a symmetric PG device at different current densities (2 to 50 mA cm^–2^). d) Areal capacitance for the symmetric PG devices at different scan rates and e) comparison of the areal capacitance of the PG electrodes with recently reported carbon-based supercapacitors (references in Table S4). f) EIS analysis on a symmetric PG device and g) a Ragone plot reporting the areal power and energy densities for carbon-based supercapacitors (references in Table S5). h) Voltage holding test on a 6-layer symmetric device, with a holding time of 30 min and (i) capacitance decay during voltage holding tests for the symmetric PG devices.

The electrochemical performance of PG electrodes was further investigated by assembling symmetric microsupercapacitors with an increasing number of layers (from 2 to 8 layers). The symmetric microsupercapacitors were assembled using identical PG electrodes with an equal working area (1 cm^2^) as positive and negative electrodes. An aqueous 1 M LiOH served as the electrolyte (Figure 5a). In agreement with the tests on three-electrode systems, the areal capacitance of the symmetric devices increases with the number of layers (Figures S13a, S10, and S11). However, the capacitance improvement between the 6- and 8-layer structures is small (1.57 F cm^–2^*vs* 1.64 F cm^–2^), as indicated by cyclic voltammetry (Figure 5d) and charge–discharge curves (Figure S13a). When gravimetric capacitances are compared, the 8-layer structures present lower performance (Figure S13a and Tables S7–S9). This might be attributed to a greater electrical transport hindrance in thicker electrodes, combined with the reduced penetration of the electrolyte into thicker structures due to longer diffusion path requirements. Thus, the 6-layer electrodes present the highest gravimetric capacitance, despite the lower mass loading. The cyclic voltammetry and charge–discharge curves for 6-layer symmetric devices are presented in Figure 5b,c. The CV curves exhibit a rectangular shape at low scan rates, with minor profile deformation at higher rates. Accordingly, the charge–discharge curves maintain a triangular shape at increasing current density, indicating the rate stability of the device. The high areal capacitance of 1.57 F cm^–2^ compares favorably with recently reported carbon-based supercapacitors (Figure 5e), and it is in line with 3D printed rGO supercapacitors,^29,35^ resulting in a superior power (0.968 mW cm^–2^) and energy density (51.2 μWh cm^–2^), as shown in the Ragone plot in Figure 5g. The gravimetric and volumetric performance are also comparable to state-of-the-art carbon supercapacitors, as demonstrated by the Ragone plots in Figure S17; nonetheless, areal properties are the most reliable metric to assess the electrochemical performance of miniaturized supercapacitors.^3^ The Nyquist plots related to the symmetric microsupercapacitors fabricated with 6-layer PG electrodes (Figure 5h) and the other electrodes (Figure S12) collectively suggest the low charge-transfer resistance and capacitive behavior of the PG electrodes. As shown in the magnified Nyquist plot (Figure 5f), the first semicircle in the high-frequency region was ascribed to the resistance of Li^+^ ion migration through the electrode. Another semicircle in the mid-frequency region was mainly produced by the kinetic resistance to charge transport (*R*_ct_), which derives from the electrolyte resistance in the porous structure of the PG electrodes, the current collector resistance, and the resistance at the electrode-current collector interface. An increase in these *R*_s_ and *R*_ct_ values could lead to the decay of the capacitance during the electrochemical tests. However, the 6-layer symmetric microsupercapacitor demonstrated lower *R*_s_ and *R*_ct_ values, indicating good electrochemical kinetics of the device. The stability of the symmetric devices was also assessed *via* voltage holding tests (VHTs), which provide insight into the degradation of the electrodes when the device is kept at the peak voltage of 1 V for prolonged times. After holding the voltage for 30 min, the electrodes were completely discharged to measure their specific capacitance and charged again to start a new voltage holding cycle (Figures 5h and S14). The printed devices show excellent capacitance retention after an overall period of several hours (Figure 5i), with almost 72% of the original capacitance retained by the 6-layer device after ∼8 h of repeated voltage holding steps. Additionally, the structure of the PG electrodes was characterized at SEM after electrochemical cycling to confirm the excellent stability provided by the PG architectures (Figure S18). The structure of the electrodes appears unaltered after 10000 cycles, presenting no cracks, delamination of the printed layers, or dissolution in the electrolyte, which would affect the electrochemical properties of the microsupercapacitors disrupting the electronic transport and reducing the amount of material effectively available for charge storage processes.

**Figure 6:**
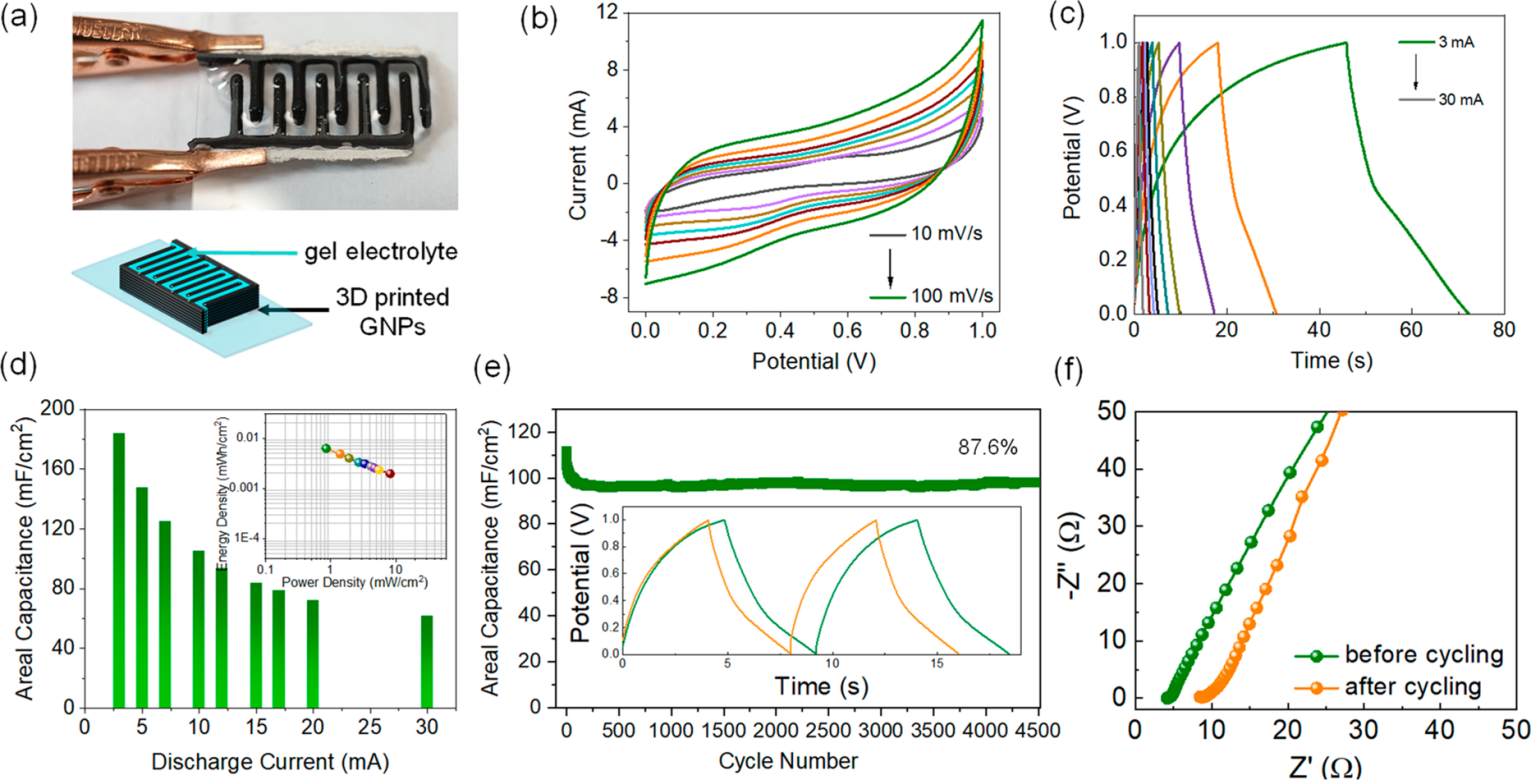
a) Picture and schematic of a quasi-solid-state interdigitated device printed on a glass substrate and filled with the gel electrolyte (footprint area ∼1.7 cm^2^). b) CV curves of the interdigitated PG device at different scan rates (10 to 100 mV s^–1^). c) GCD on the interdigitated PG at different currents (3 to 30 mA) and d) capacitance retention of the interdigitated device at an increasing discharge current. The inset in d) shows a Ragone plot for the interdigitated device. e) Cycling test on the interdigitated device at a current of 10 mA (inset shows the first two and last two charge–discharge curves, in green and orange, respectively). f) EIS on the interdigitated device before and after cycling.

The suitability of the PG ink for the 3D printing of energy devices was further demonstrated *via* the fabrication of interdigitated microsupercapacitors on glass substrates, which were tested as quasi-solid-state devices. They did not require a prepatterned current collector thanks to the high conductivity of the PG platelets. Two 6-layer interdigitated electrodes (Figures 6a and S16) were simultaneously deposited on the substrate and subsequently annealed to remove the binders from the ink. A PVA-LiOH gel electrolyte was then injected between the electrode fingers. The electrochemical properties of the interdigitated PG device were studied *via* cyclic voltammetry (Figure 6b) and galvanostatic charge–discharge (Figure 6c). Despite the absence of the current collector, the microsupercapacitor was able to deliver an areal capacitance of 184 mF cm^–2^, which is superior to similar interdigitated devices obtained *via* the DIW of rGO (74.31 mF cm^–2^)^29^ and activated carbon (104.4 mF cm^–2^).^52^ The rate capability and cycling stability of the interdigitated microsupercapacitor are presented in Figure 6d,e, exhibiting a stable cycling behavior after an initial capacitance loss of ∼10% in the first hundreds of cycles. The equivalent series resistance of the interdigitated microsupercapacitor was calculated from the internal resistance (IR) drop in the charge–discharge curves and from electrochemical impedance spectroscopy (Figure S16f), leading to comparable values of resistance, *i.e*., 5.18 Ω and 4.33 Ω before cycling, respectively, and 10.04 Ω and 9.04 Ω after cycling, respectively. The device resistance is usually attributed to the charge transport resistance inside the electrodes and the electrolyte and to the contact resistance at the interface with the current collector. The absence of a current collector and the long transport distance inside 3D electrodes determine a larger series resistance than in thin-film carbon supercapacitors.^53^ Nonetheless, the resistance of the interdigitated microsupercapacitor compares favorably with similar 3D printed carbon devices recently reported (32.9 Ω),^54^ despite the absence of a current collector. The increase in internal resistance observed after cycling can be attributed to the evaporation of water from the gel electrolyte over extended periods of time, which decreases the ionic mobility.^55^ The internal resistance of the device, combined with the small stability window of aqueous gel electrolytes, represents the main causes for the limited Coulombic efficiencies reported in Figure S16e.^56^ Higher efficiencies could be achieved by encapsulating the device to avoid the detrimental increase in internal resistance over cycling; additionally, replacing the aqueous gel electrolyte with ionic liquids or water-in-salt electrolytes can enhance the stability and hence the Coulombic efficiency of the interdigitated supercapacitors.^57,58^

## Conclusions

A nearly one-step manufacturing of all-carbon three-dimensional microsupercapacitors based on PG aqueous inks was here demonstrated. The as-printed 3D PG electrodes present prime electrical conductivity and high accessible surface area, resulting in current collector-free devices with rapid charge transfer paths and rich reservoirs for ion absorption during the energy storage process. The printed microsupercapacitors exhibit a maximum cell voltage of 1.0 V with a capacitance of 1.57 F cm^–2^, which are competitive with respect to previously reported 3D printed carbon devices. Additionally, the stability tests and kinetic analysis confirmed the excellent performance of our PG-based devices, indicating superior voltage holding capability and fast charge transport in the electrodes. The printed microsupercapacitors also demonstrated maximum areal energy and power densities of 51.2 μWh cm^–2^ and 0.968 mW cm^–2^, respectively. In summary, this work showed how the intrinsic properties of PG platelets can be exploited to make efficient devices and how the vertical structuring of electrodes over small footprint areas allows achievement of prime areal capacitance to power on-chip devices.

## Experimental Section

### Graphene Characterization

PG platelets (5 μm particle size, Sigma-Aldrich) were used as-received. Powder X-ray diffraction was performed on the PG platelets and on printed structures with a Bruker D2 Phaser diffractometer equipped with a copper source in the range 2–80° for 2θ and with an angular step size of 0.034°. The printed structures were manually ground in a mortar before XRD analysis. Raman spectra were acquired using a Renishaw inVia Qontor confocal Raman microscope at a wavelength of 532 nm and using a laser power of 0.5 mW. The PG platelets were dispersed in ethanol and drop-casted on Si/SiO_2_ substrates before collecting Raman spectra. PG platelets were imaged using a JEOL JEM-2100F TEM with a field emission gun operated at 200 kV accelerating voltage. The TEM samples were prepared dispersing the PG platelets in ethanol and drop casting the dispersion onto TEM grids. For optical imaging, the PG platelets were dispersed in ethanol and drop-casted on Si/SiO_2_ substrates. For AFM characterization, PG platelets were dispersed in ethanol with a concentration of 0.1 mg/mL. Ten microliters of the dispersion was drop-casted on a Si wafer and kept for 15 min for fully drying. AFM imaging was performed in air tapping mode by using an oxide-sharpened silicon nitride AFM tip (Veeco Inc., CA, USA). AFM images were obtained by a Digital Instruments Nanoscope V8 (Bruker, MA, USA).

### Ink Preparation and Characterization

To formulate the PG inks, sodium carboxymethyl cellulose (average Mw ∼ 250,000, degree of substitution 1.2, Sigma-Aldrich) was dissolved in distilled water (4 wt %) before adding the graphene platelets to the solution. The graphene dispersion was then homogenized using a planetary mixer (Thinky ARE-250) at 1800 and 2000 rpm for 10 min. 1-octanol (≥98%, FCC, FG, Sigma-Aldrich) was added stepwise into the graphene dispersion to a final concentration of ∼2 vol %, homogenizing the slurry at 2000 rpm after each addition. The ink was stored in a closed jar and rehomogenized before use. The rheology of the PG ink was measured using a rotational rheometer (Discovery Hybrid Rheometer HR1 – TA Instruments) equipped with stainless-steel parallel plates (40 mm in diameter). To avoid wall slip during the flow tests, two discs of 600-grit sandpaper were attached to the rheometer plates. A solvent trap was also employed during the measurements to minimize water evaporation from the samples. The test temperature was kept at 25 °C with a Peltier plate. The flow and oscillatory measurements were performed three times using different samples, and the instrument was cleaned and recalibrated after each test. Oscillatory stress sweeps were performed at a fixed frequency of 1 Hz, while oscillatory recovery tests were performed at 10 Hz to maximize the acquisition of points over time. Oscillatory frequency sweeps were conducted at a fixed strain amplitude of 0.05%, within the linear viscoelastic region for the PG inks, as determined from the oscillatory stress sweeps. To measure the electrical conductivity of the PG ink, the ink was slurry-coated onto an insulating substrate (microscope glass slide) using a glass rod. The slurry-coated film was then contacted with conductive silver paint, and the I–V curve of the material was measured using a Gamry Interface 1000 galvanostat (4-probe measurement). The electrical conductivity was calculated from the I–V curve as detailed in the Supporting Information.

### 3D Printing and Electrode Characterization

The PG inks were loaded into 3 mL polypropylene syringes with blunt nozzles (inner diameter between 200 and 410 μm) just before printing. The syringes were mounted onto the three-axis stage of the 3D printer and connected to displacement-controlled plungers, which provided a feed rate of 6 mm s^–1^. Woodpile electrodes (2 to 8 layers) were printed on graphite foil for three-electrode and two-electrode electrochemical measurements. After printing, the structures were dried in air overnight and then annealed in a tubular furnace at 350 °C for 30 min (Ar atmosphere ∼ 0.5 mbar). Scanning electron microscopy (SEM) images of the uncoated structures were collected with a Zeiss Auriga SEM operated at an accelerating voltage of 3 kV (working distance ∼ 5 mm). The three-dimensional microstructure of the printed filaments was investigated using X-ray nanocomputed tomography (Zeiss Xradia 810 Ultra, Carl Zeiss X-ray Microscopy Inc., Pleasanton, CA) in a large-field-of-view mode (65 μm width) acquiring phase-contrast images. The central region of the filament was scanned using binning 1 (63 nm voxel resolution), while the outer region was scanned using binning 2 (126 nm voxel resolution). The porosity of the filament was calculated from the scan in the central region, segmenting the graphene platelets with Avizo software (Thermo Fisher Scientific, USA) and using FiJi (ImageJ) to compute the local thickness and to approximate a sphere occupancy in the void space. The orientation of platelets was evaluated using the OrientationJ plugin for ImageJ (FiJi), based on the calculation of the gradient structure tensor in a local neighborhood of the image. The orientation analysis was performed on all the slices normal to the filament axis, and the results were summed and normalized to obtain the orientation histogram.

### Electrochemical Testing

Three-electrode tests were performed in 1 M LiOH using a commercial Ag/AgCl reference electrode and graphite foil (∼1 cm^2^ area) as a counter electrode. Woodpile structures were also used to assemble symmetric devices in 1 M LiOH, which were tested in a two-electrode configuration. Both the electrodes of each device consisted of the same number of printed layers and had nearly the same mass. Cyclic voltammetry (CV) and galvanostatic charge–discharge (GCD) curves were acquired in a potential window of 1 V at different scan rates and current densities using a Gamry 1000 Interface potentiostat. The specific capacitance, power, and energy of the devices were calculated from CV and GCD curves as detailed in the Supporting Information. Electrochemical impedance spectroscopy (EIS) was performed between 0.01 Hz and 100 kHz at an AC voltage of 5 mV rsm.

### Preparation of the LiOH Gel Electrolyte

Mowiol 56-98 (Poly(vinyl alcohol) Mw ∼ 195000, Sigma-Aldrich) was dissolved in deionized water stirring continuously at ∼90 °C. After complete dissolution, LiOH (anhydrous, 98%, Alfa Aesar) was added to the polymer solution. The weight ratio of water, polyvinyl alcohol, and LiOH was fixed at 20:2:1. The gel electrolyte was then loaded into a syringe and stored at room temperature.

### Interdigitated Device Assembly

Interdigitated electrodes were printed on microscope glass slides and dried in air overnight. The electrodes were subsequently annealed at 350 °C in Ar for 30 min, and then they were contacted using silver paint. The LiOH gel electrolyte was injected between the interdigital electrodes using a syringe, avoiding contact with the silver paint.

## Supporting Information Available

Raman, XRD, and TEM characterization of PG platelets; index of printability; rheological recovery tests; detailed calculation of plug-flow radius; detailed characterization of electrical conductivity; cyclic voltammetry and galvanostatic charge–discharge at different scan rates and current densities; detailed calculation of electrochemical properties (PDF)
