# Supplementary material for: Aqueous Inks of Pristine Graphene for 3D Printed Microsupercapacitors with High Capacitance
Source: ACS Nano. 2021 Sep 7;15(9):15342–53. doi: 10.1021/acsnano.1c06535 (PMC8482754; doi:10.1021/acsnano.1c06535)
Supplement: Supplementary file 1 — nn1c06535_si_001.pdf [file nn1c06535_si_001.pdf]

SUPPORTING INFORMATION

Aqueous Inks of Pristine Graphene for 3D Printed Microsupercapacitors with High Capacitance

Stefano Tagliaferri,[a] *Goli Nagaraju,* [a] *Apostolos Panagiotopoulos,* [a] *Mauro Och,* [a] *Gang Cheng,* [a] *Francesco Iacoviello,*[b] *Cecilia Mattevi** [a]

[a] Department of Materials, Imperial College London, London SW7 2AZ, United Kingdom

[b] Electrochemical Innovation Lab, Department of Chemical Engineering, University College London, London WC1E 7JE, UK

*Corresponding author: c.mattevi@imperial.ac.uk

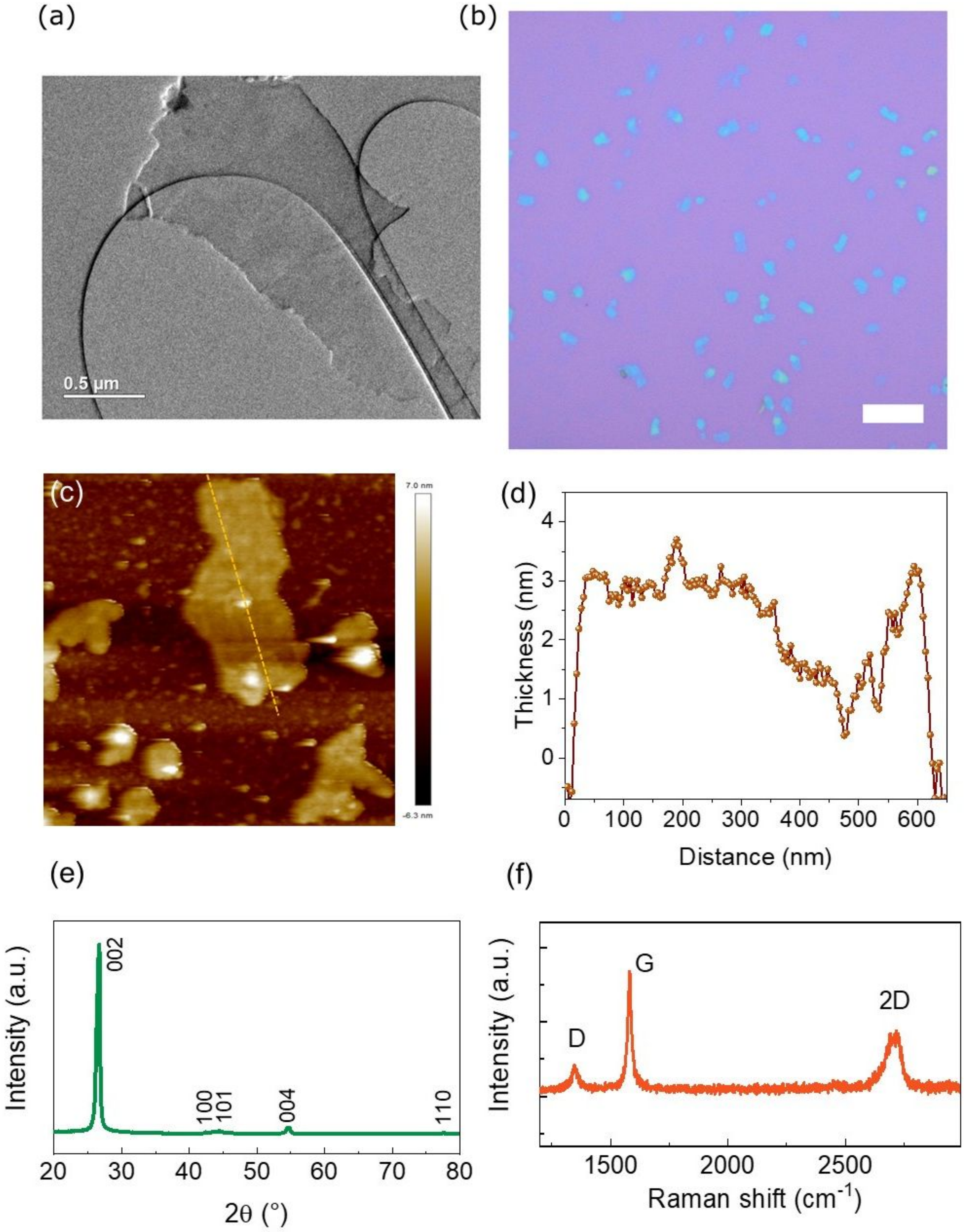

FIGURE S1: a) TEM image of the graphene nanoplatelets used in this work; b) Optical image of graphene nanoplatelets on a Si/SiO_2 substrate (scalebar 10µm); c,d) AFM analysis of the graphene platelets used in this work (lateral side of the picture in (c) is 1 µm) and corresponding profile thickness, showing that the graphene platelets consist of ~10 layers; e.f) X-Ray Diffraction (XRD)

and Raman spectroscopy on the pristine PG platelets. The sharp peaks in the XRD pattern highlight the good crystallinity of the PG platelets and can be indexed as graphitic carbon, confirming the phase purity of the graphene material. The Raman spectrum exhibits the characteristic features of graphitic materials: G-band (1581 cm^{-1}), associated with the E_{2g} in-plane phonon mode at the Brillouin zone centre, D-band (1348 cm^{-1}), activated by the breathing mode of sp^2 atoms and 2D-band (2705 cm^{-1}), activated by second-order phonons.[1,2] A small shoulder is also present at ~1617 cm^{-1}, partially convoluted with the G band, which can be assigned to the graphene D' band. The position close to 1580 cm^{-1} and the large FWHM (~18 cm^{-1}) of the G band, together with the small I(D)/I(G) ratio (~0.18), are indicative of the chemical purity and low number of defects in the graphene sample.[1,3] Additionally, the shape of the 2D band and the position of the G-band denote the multi-layer (>10 layers) nature of the PG platelets used.[4,5]

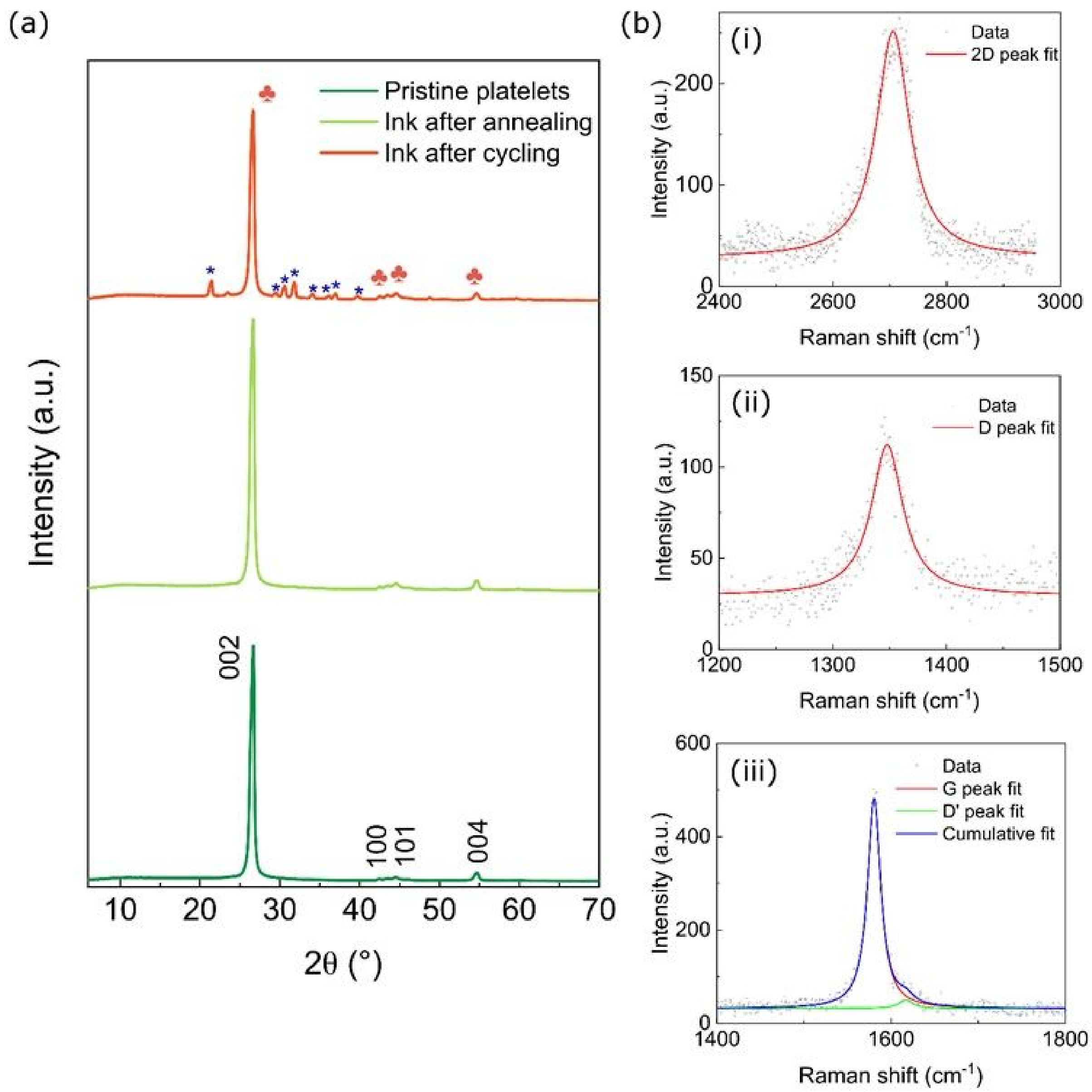

FIGURE S2: a) Powder X-ray diffraction pattern of the pristine graphene platelets, ink after annealing and printed electrodes after electrochemical cycling (the peaks labelled with a blue asterisk can be attributed to Li_2CO_3, possibly forming from the electrolyte during drying in air). The XRD pattern remains unchanged after ink formulation and annealing, indicating that the crystalline structure of PG platelets is stable during ink processing. b) Lorentzian fittings of the 2D (i), D (ii) and G+D' (iii) bands in PG Raman spectrum.

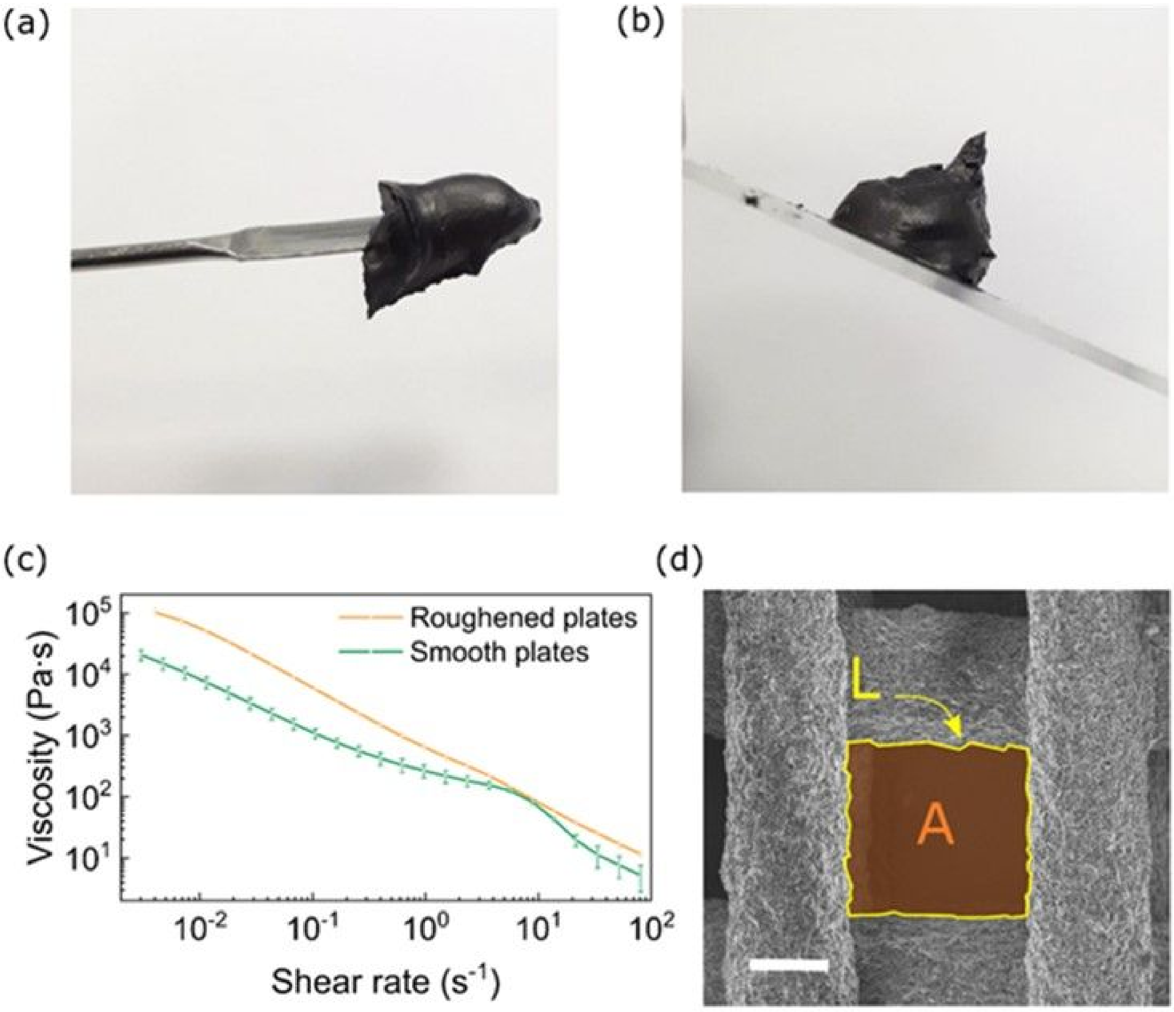

FIGURE S3: a,b) Pictures of the PG ink after high-speed homogenization: the ink appears smooths and it is able to self-stand, owing to its yield-stress rheology; c) Flow curves measured using smooth and roughened plates: when smooth plates are used flow instabilities and ejection of material are more likely to occur, causing an apparent reduction of the shear stress with increasing shear rate and the appearance of a “bump” in the viscosity curve. The viscosity is also significantly underestimated, and the rheological measurements are less reproducible when smooth plates are used. d) Schematic showing how the index of printability can be calculated from SEM images of the woodpile electrodes (see Equation S1). The scalebar is 200 µm.

TABLE S1: Results of the Herschel-Bulkley fitting on the flow curve (shear stress *vs*. shear rate) in the range 10^{-3} - 10^{2} s^{-1}

τ_y (Pa)	$\dot{\gamma}_c$ (s^{-1})	*n*	*R-Square*
491	45.8	0.430	0.986

TABLE S2: Yield stress and storage modulus for literature-reported inks formulated to print energy storage devices *via* DIW.

Material	Yield Stress (Pa)	Storage Modulus (kPa)	Ref.
MXenes	24	20	[6]
MXenes	206	36	[7]
GO+Ca^{2+}	160	60	[8]
holey GO	500	30	[9]
GO	50	3	[10]
GO+urea+GδL	450	40	[11]
PEDOT:PSS	200	2	[12]
Gallium alloy	95	2	[13]
CNF	300	5	[14]
PG graphene	58	102	this work

Index of printability: The index of printability (Pr) was used to quantify the shape retention of printed structures. It was calculated from SEM images of woodpile electrodes (Figure S3d) as:[15]

$$Pr = \frac{L^2}{16A} \# (\textbf{Equation S1})$$

where L is the perimeter of one of the woodpile grid holes (Figure S3d) and A is its area. If the grid hole is a perfect square (ideal shape retention) Pr =1.

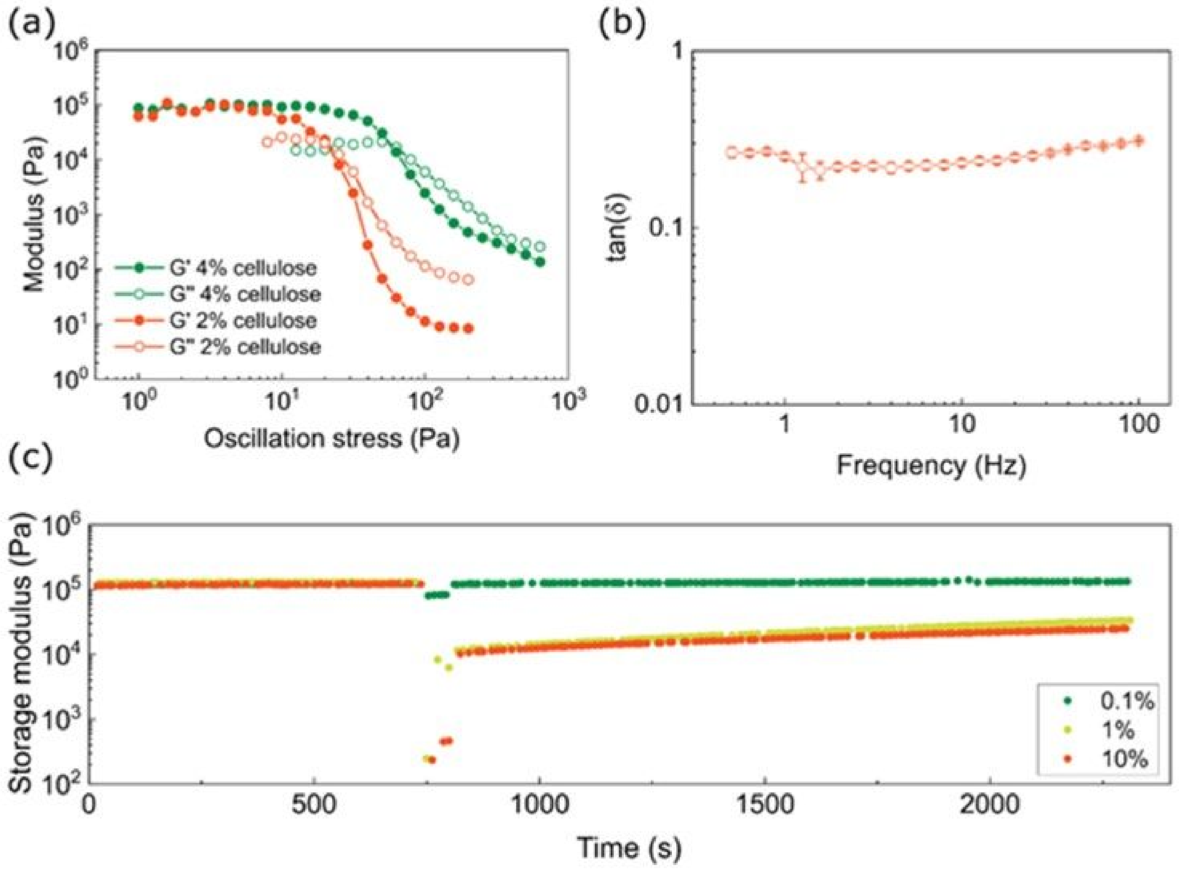

FIGURE S4: a) Comparison between the oscillatory response of PG inks containing 2% and 4% cellulose. A reduction in the amount of cellulose does not significantly alter the storage modulus, whereas the yield stress becomes threefold smaller. Additionally, the graphene platelets are not stable in the 2% ink, causing frequent clogging of the printing nozzle. b) Loss tangent as a function of frequency in the linear viscoelastic region for the PG inks; c) Extended three-interval thixotropy tests at different oscillatory strain amplitudes during the destruction step (0.1%, 1%, 10% for 60 s, respectively). After 1% and 10% steps the storage modulus does not fully recover to the initial value, although it is still able to rapidly revert to $\sim 10^4$ Pa.

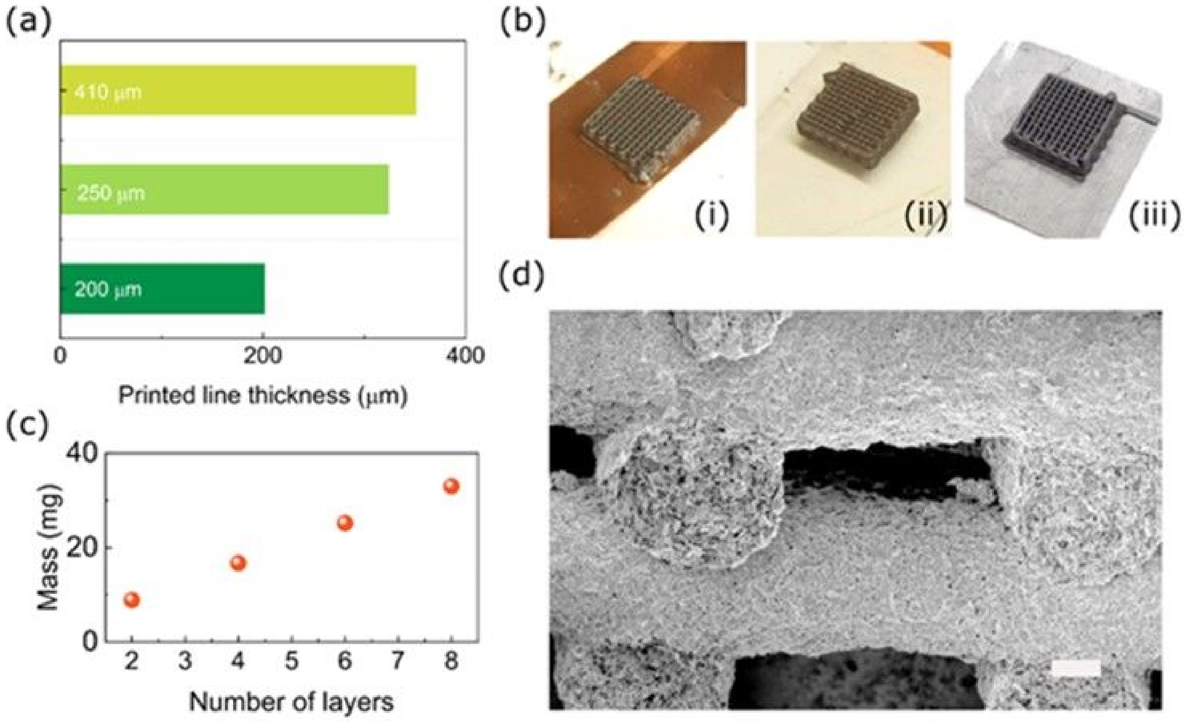

FIGURE S5: a) Comparison of the thickness of printed structs fabricated with 200-, 250-, and 410-µm nozzles, respectively; b) Pictures showing printed woodpile structures directly fabricated on copper foil (i), ITO-coated glass (ii) and graphite foil (iii); c) Mass of the woodpile structures as a function of the number of printed layers; d) SEM picture of the cross-section of a printed woodpile structure (scalebar 100 µm)

Calculation of the plug-flow radius: To calculate the radius of the plug flow region the ink was modelled as a Herschel-Bulkley fluid flowing in a cylindrical nozzle. For such a system, the radius of the inner region that experiences plug flow is given by:

$$R_p = \frac{\tau_y}{\tau_w} R = \varphi R \#(\textbf{Equation S2})$$

where R_p is the radius of the plug flow region, *R* the radius of the nozzle, τ_w the stress at the wall of the nozzle (maximum stress) and φ the ratio between the yield stress and the stress at the wall. φ was calculated from the following implicit expression using Matlab:[16]

$$V = nR\left(\frac{\tau_y}{K\,\varphi}\right)^{1/n}(1-\varphi)^{\frac{n+1}{n}}\left(\frac{(1-\varphi)^2}{3n\;+1}+\frac{2\varphi(1-\varphi)}{2n\;+1}+\frac{\varphi^2}{n\;+1}\right)\#(\textbf{Equation S3})$$

where *n, K* and τ_y are the Herschel-Bulkley parameters obtained by fitting the flow curve of the PG inks with the Herschel-Bulkley model, *V* is the extrusion rate (6 mm s^{-1}) and *R* is the nozzle radius

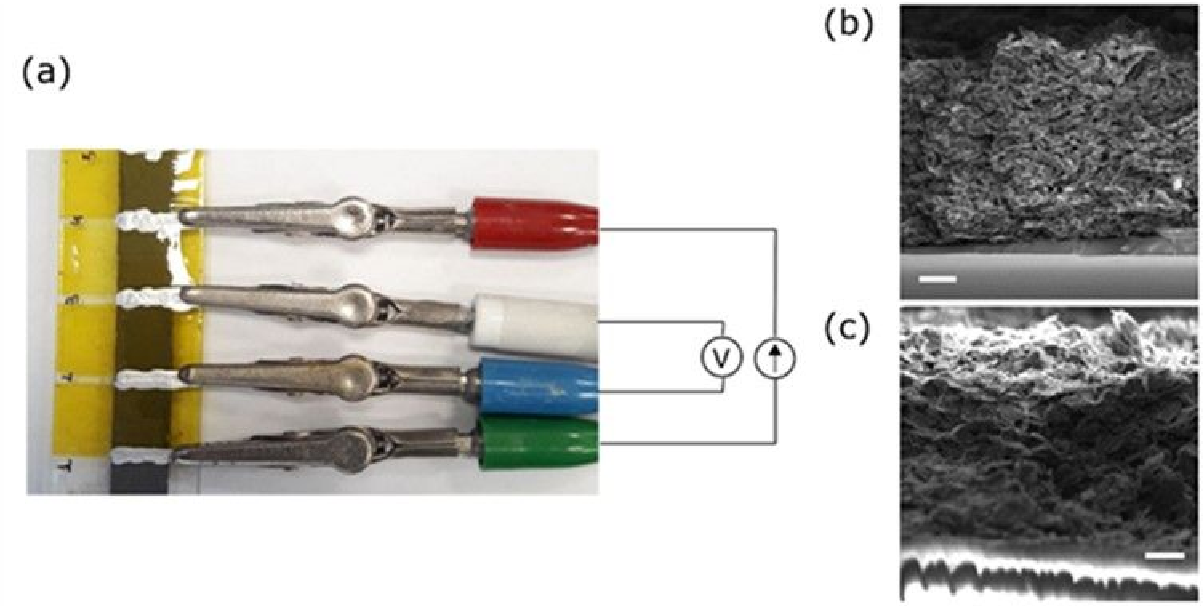

FIGURE S6: a) Schematic of the 4-electrode tests performed to determine the conductivity of slurry-coated films obtained from the PG inks. A sawtooth current bias was applied between the working (green) and counter (red) electrodes and the potential between the working sense (blue) and reference (white) electrodes was measured. b,c) SEM images showing the thickness of the films used to measure the conductivity of PG inks: (b) film before annealing (scalebar 30 μm) and (c) film after annealing (scalebar 20 μm)

Electrical conductivity: I-V curves were measure in a four-electrode configuration on a slurry-coated film obtained from the PG ink (Figure S6). A sawtooth current signal was applied between terminal 1 and 4 and the potential between terminal 2 and 3 was recorded. The conductivity of the ink was calculated according to:

$$\sigma = \frac{L}{W \cdot H \cdot R} \# (\textbf{Equation S4})$$

where R is the resistance (obtained from a linear fitting of the I-V curve), L is the distance between terminal 2 and 3, W is the width of the film and H is the thickness of the film (measured using SEM, Figure S6).

Table S3: Comparison of the electrical conductivity of DIW inks

**Material**	**Conductivity (S m^{-1})**	**Reference**
PG ink (annealed)	1372	This work
PG ink (not annealed)	268	This work
rGO (1)	800	[8]
rGO (2)	170	[10]
rGO (3)	40	[17]

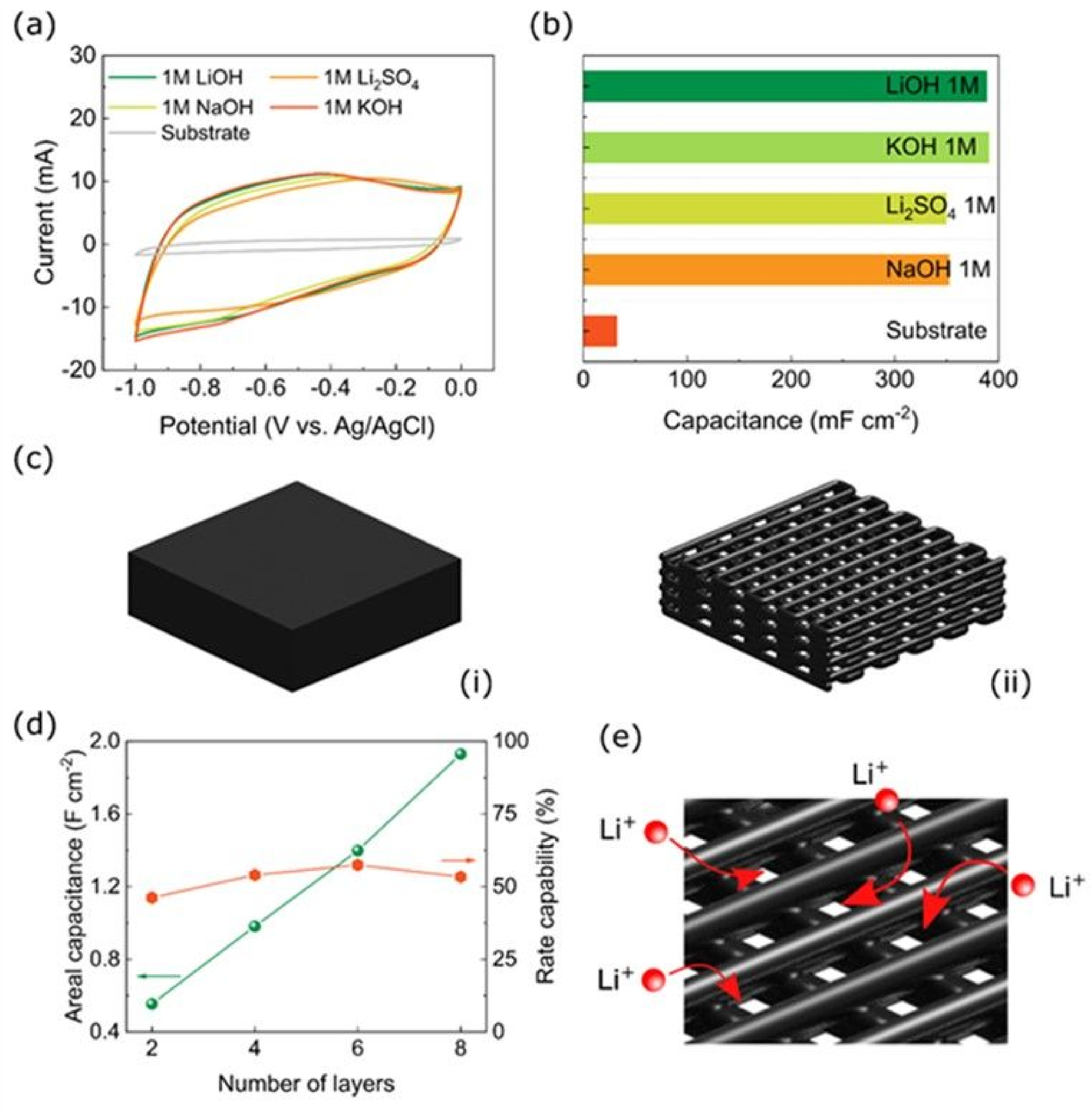

FIGURE S7: a) Plot comparing the cyclic voltammograms of a woodpile electrode in different electrolytes (LiOH, NaOH, KOH, Li_2SO_4) and the cyclic voltammogram of the bare substrate (graphite foil) in LIOH 1M.; b) Comparison between the capacitance of a woodpile electrode in different electrolytes and the bare substrate in LiOH 1M; c) Schematics of a compact (i) and woodpile (i) structure; d) Plot comparing the areal capacitance and rate capability for different woodpile electrodes with increasing thickness; e) Schematic showing how the channels in the woodpile structures can facilitate the penetration of the electrolyte, improving the surface area accessible to Li^+ ions

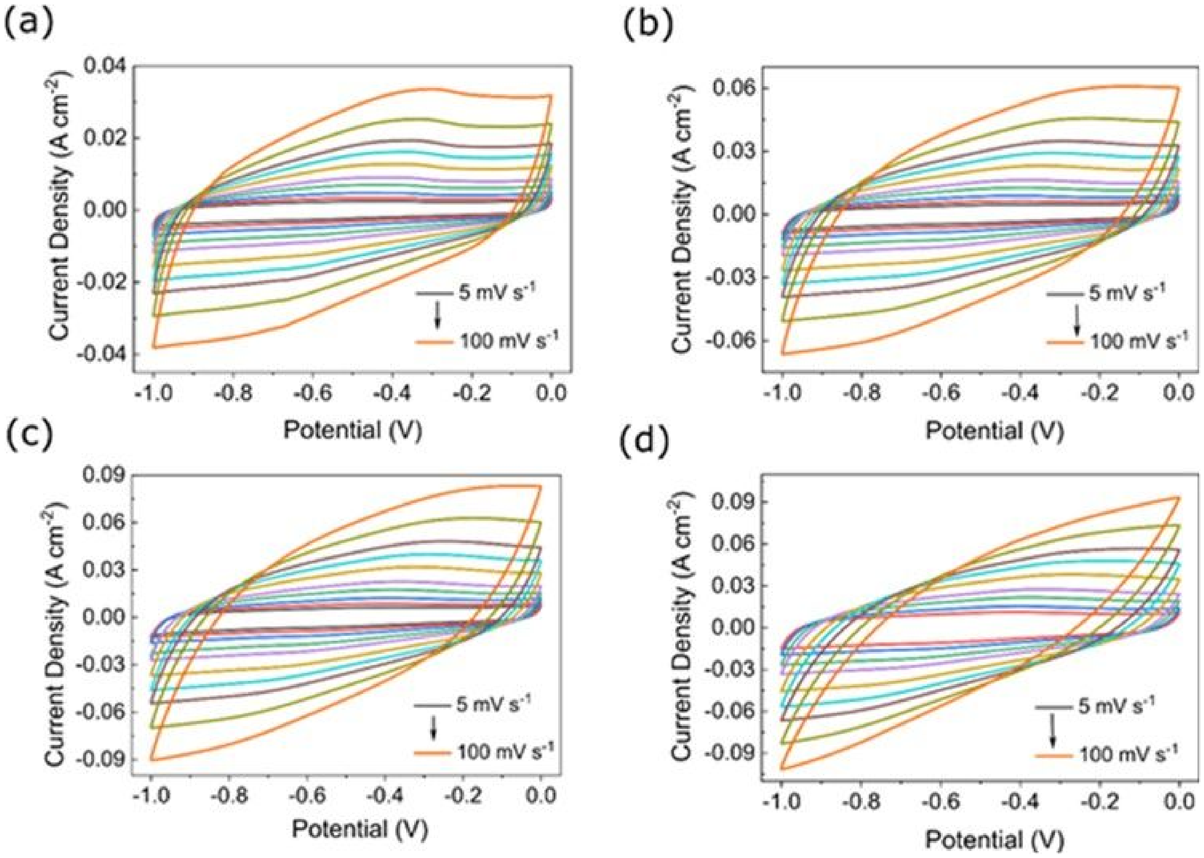

FIGURE S8: a-d) Cyclic voltammograms of 2-layer (a), 4-layer (b), 6-layer (c) and 8-layer (d) electrodes tested in a three-electrode configuration at different scan rates (5, 7, 10, 15, 20, 30, 40, 50, 70 and 100 mV s^{-1})

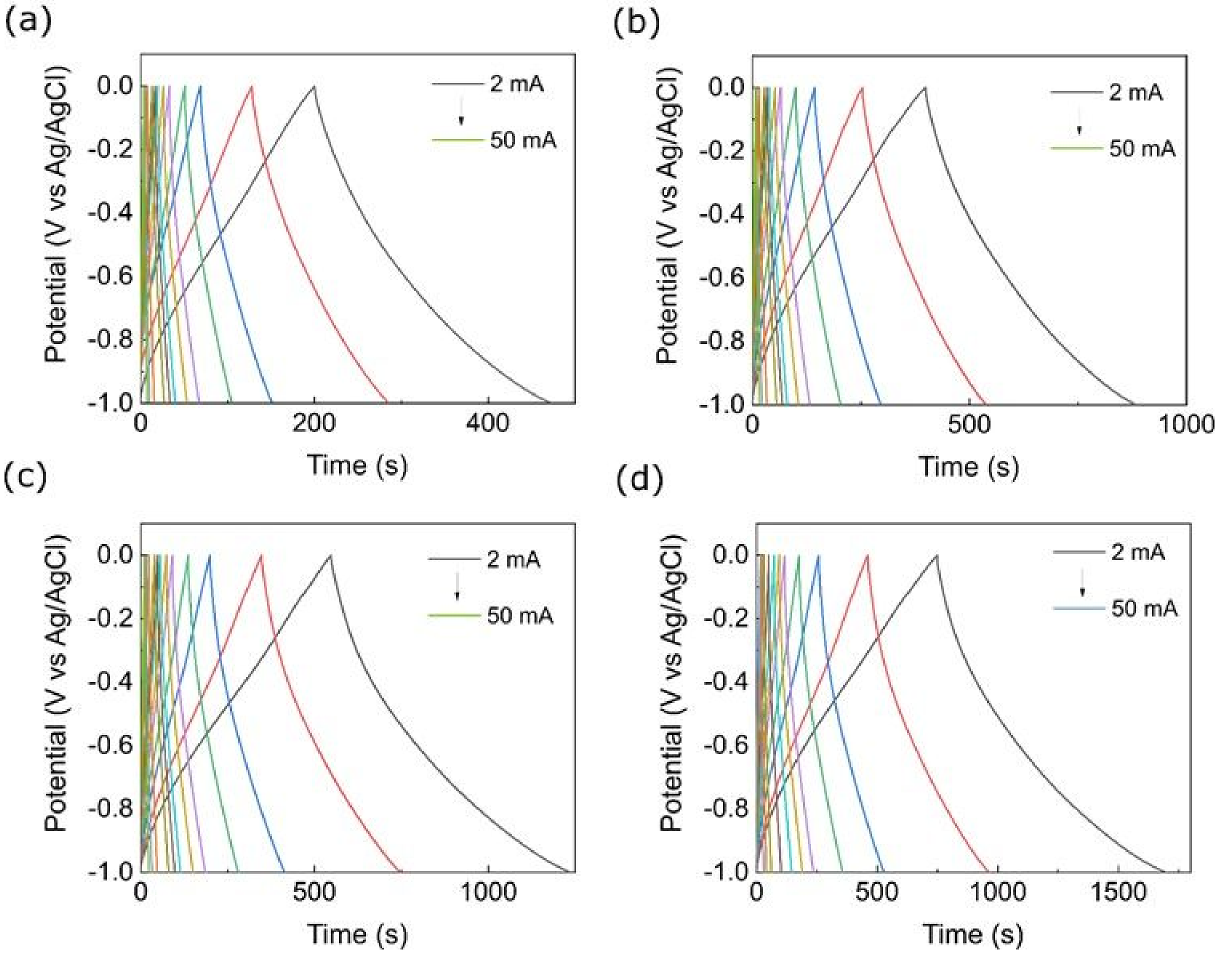

FIGURE S9: a-d) Galvanostatic charge- discharge curves of 2-layers (a), 4-layer (b), 6-layer (c) and 8-layer (d) electrodes tested in a three-electrode configuration at different current densities (2, 3, 5, 7, 10, 12, 15, 17, 20, 30, 40, 50 mA cm^{-2})

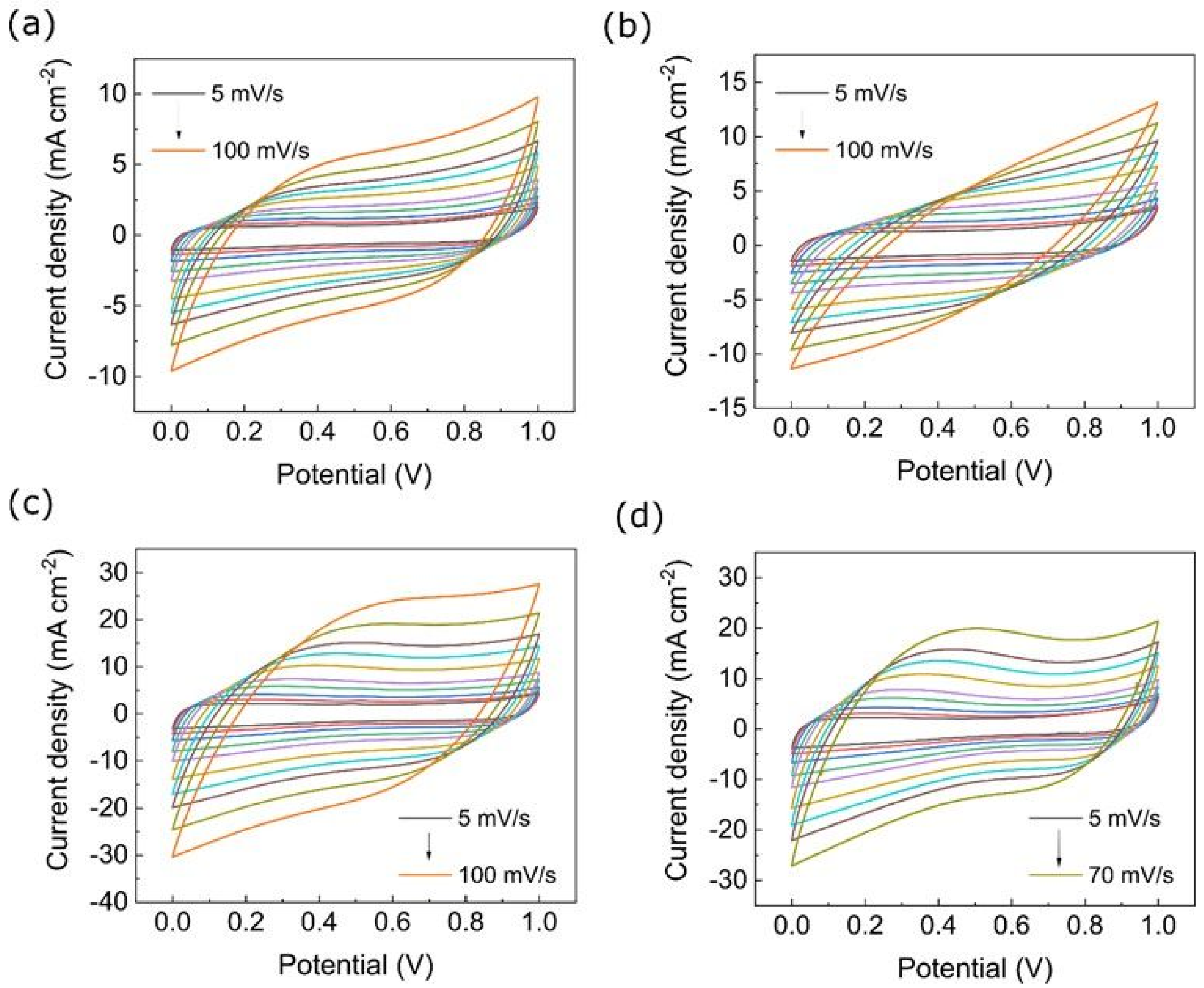

FIGURE S10: a-d) Cyclic voltammograms of 2-layer (a), 4-layer (b), 6-layer (c) and 8-layer (d) symmetric devices at different scan rates (5, 7, 10, 15, 20, 30, 40, 50, 70 and 100 mV s^{-1})

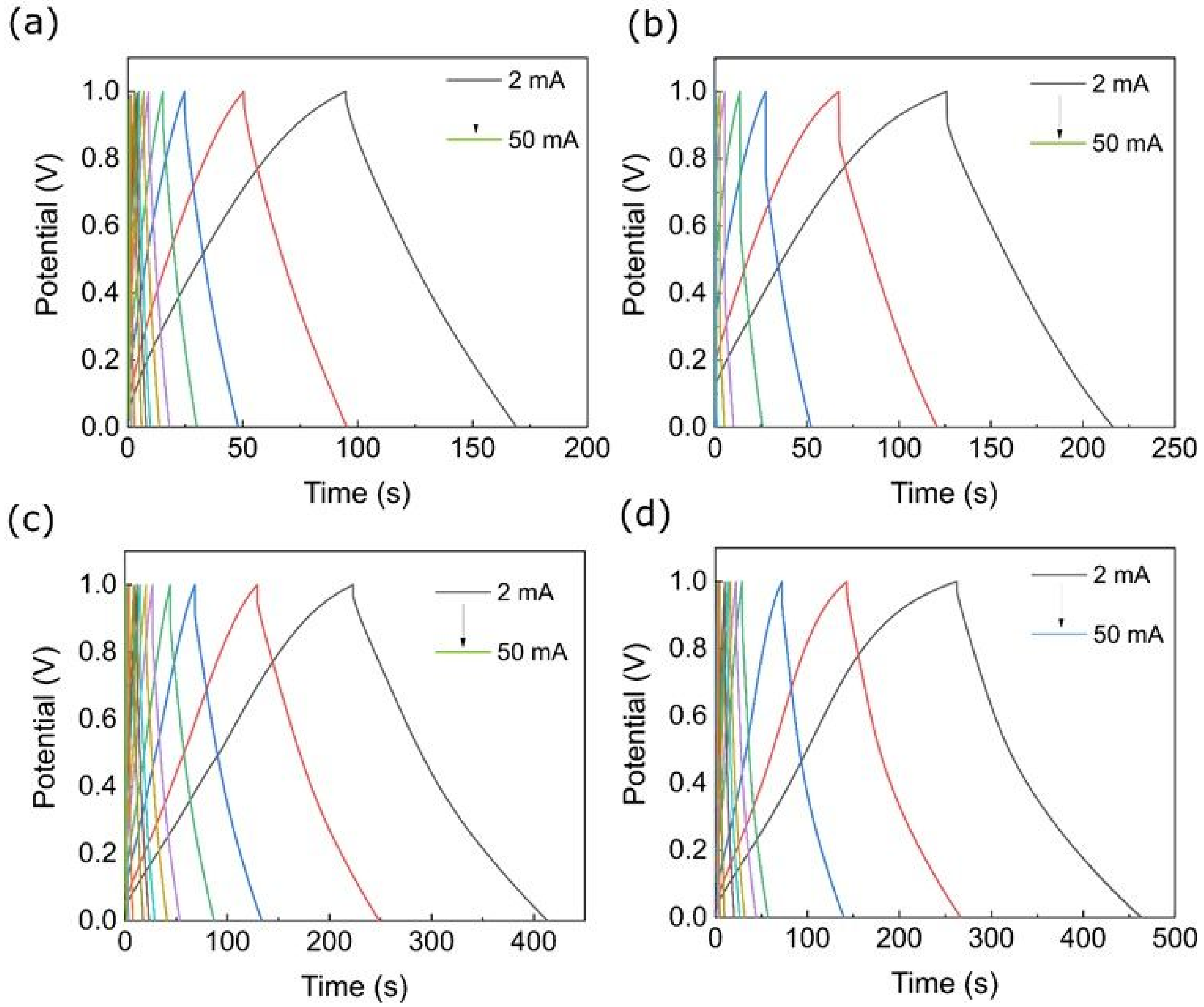

FIGURE S11: a-d) Galvanostatic charge- discharge curves of 2-layers (a), 4-layer (b), 6-layer (c) and 8-layer (d) symmetric devices at different current densities (2, 3, 5, 7, 10, 12, 15, 17, 20, 30, 40, 50 mA cm^{-2})

TABLE S4: Comparison of areal capacitances reported in recent literature for supercapacitors

**Material**	**Areal capacitance**	**Ref.**
MXenes	43 mF cm^{-2}	[18]
IJP graphene	99 μF cm^{-2}	[19]
Spray-coated graphene	1080 μF cm^{-2}	[20]
rGO (blue)	323 μF cm^{-2}	[21]
Laser-reduced GO	0.51 mF cm^{-2}	[22]
S-doped graphene	553 μF cm^{-2}	[23]
Photoreduced graphene	1.5 mF cm^{-2}	[24]
Cellular rGO films	2.47 mF cm^{-2}	[25]
G/CNTs	3.93 mF cm^{-2}	[26]
rGO	6.1 mF cm^{-2}	[27]
CDC	205 mF cm^{-2}	[28]
rGO hydrogel film	402 mF cm^{-2}	[29]
rGO film	71 mF cm^{-2}	[30]
rGO film	246 mF cm^{-2}	[31]
3D GA	640 mF cm^{-2}	[17]
PG inks	1.64 F cm^{-2}	This work

TABLE S5: Comparison of areal energy and power densities reported in recent literature for carbon-based supercapacitors

**Material**	**Power density ($mW\ cm^{-2}$)**	**Energy density ($\mu Wh\ cm^{-2}$)**	**Reference**
Coaxial carbon fibers	0.191<br>0.466<br>0.899<br>1.92<br>4.55<br>8.16	9.75<br>8.20<br>7.08<br>5.88<br>3.18<br>1.48	32
rGO film (purple)	22.2<br>35.5<br>48.2<br>70.0<br>118	5.56<br>5.20<br>4.66<br>4.35<br>3.50	31
rGO film (yellow)	0.486<br>0.983<br>1.923<br>2.84<br>3.85<br>4.75<br>9.19	9.78<br>9.44<br>8.87<br>8.74<br>8.61<br>8.43<br>7.76	30
rGO hydrogel	5.05<br>2.48<br>1.02<br>0.538<br>0.538	10.2<br>11.2<br>12.3<br>12.9<br>12.9	29
CDC	0.286<br>0.546<br>1.11<br>2.55<br>4.71<br>8.83<br>19.2<br>33.8	1.60<br>1.55<br>1.47<br>1.39<br>1.30<br>1.22<br>1.07<br>0.916	28
rGO + CNTs (orange)	1.52<br>4.04<br>6.52<br>12.2	0.406<br>0.381<br>0.359<br>0.333	27
G/CNTs	1.00<br>2.50<br>4.98<br>9.75<br>22.7<br>40.8<br>61.8	0.292<br>0.286<br>0.274<br>0.257<br>0.222<br>0.170<br>$9.41 \cdot 10^{-2}$	26
3D GA	1.52	20.5	17

3D printed GA	$7.43\cdot10^{-5}$<br>$1.18\cdot10^{-4}$<br>$1.58\cdot10^{-4}$<br>$2.58\cdot10^{-4}$<br>$4.10\cdot10^{-4}$<br>$8.26\cdot10^{-4}$<br>$1.31\cdot10^{-3}$<br>$2.64\cdot10^{-3}$<br>$3.99\cdot10^{-3}$	17.0<br>18.6<br>19.2<br>21.0<br>22.9<br>25.0<br>26.2<br>27.6<br>28.2	[33]
Cellular rGO films	$4.10\cdot10^{-3}$<br>$29.8\cdot10^{-3}$<br>$67.0\cdot10^{-3}$<br>0.122<br>0.206<br>0.274<br>0.340<br>0.393	0.222<br>0.190<br>0.175<br>0.154<br>0.127<br>0.111<br>$9.64\cdot10^{-2}$<br>$8.90\cdot10^{-2}$	[25]
Photoreduced graphene	1.97<br>0.521	$4.85\cdot10^{-2}$<br>$9.45\cdot10^{-3}$	[24]
S-doped graphene	1.19	$3.10\cdot10^{-3}$	[23]
Laser reduced-GO	1.55	$3.13\cdot10^{-2}$	[22]
rGO (blue)	$3.83\cdot10^{-4}$<br>$6.45\cdot10^{-4}$<br>$1.49\cdot10^{-3}$<br>$2.41\cdot10^{-3}$<br>$4.33\cdot10^{-3}$<br>$10.5\cdot10^{-3}$<br>$19.8\cdot10^{-3}$<br>$37.9\cdot10^{-3}$<br>$86.5\cdot10^{-3}$<br>0.133<br>0.159<br>0.278<br>0.379<br>0.539	$1.13\cdot10^{-2}$<br>$9.21\cdot10^{-3}$<br>$8.48\cdot10^{-3}$<br>$6.92\cdot10^{-3}$<br>$6.28\cdot10^{-3}$<br>$5.95\cdot10^{-3}$<br>$5.63\cdot10^{-3}$<br>$5.33\cdot10^{-3}$<br>$4.84\cdot10^{-3}$<br>$4.59\cdot10^{-3}$<br>$4.40\cdot10^{-3}$<br>$3.89\cdot10^{-3}$<br>$3.49\cdot10^{-3}$<br>$3.00\cdot10^{-3}$	[21]
S-graphene	$5.07\cdot10^{-4}$<br>$1.76\cdot10^{-3}$<br>$3.65\cdot10^{-3}$	0.109<br>$9.00\cdot10^{-2}$<br>$8.71\cdot10^{-2}$	[20]

	$5.79 \cdot 10^{-3}$<br>$11.2 \cdot 10^{-3}$<br>$14.6 \cdot 10^{-3}$<br>$16.5 \cdot 10^{-3}$	$7.39 \cdot 10^{-2}$<br>$6.16 \cdot 10^{-2}$<br>$5.03 \cdot 10^{-2}$<br>$4.52 \cdot 10^{-2}$	
IJP graphene	$4.09 \cdot 10^{-5}$<br>$8.03 \cdot 10^{-5}$<br>$1.91 \cdot 10^{-5}$<br>$3.65 \cdot 10^{-4}$<br>$6.81 \cdot 10^{-4}$<br>$1.47 \cdot 10^{-3}$<br>$2.68 \cdot 10^{-3}$<br>$4.93 \cdot 10^{-3}$	$2.22 \cdot 10^{-3}$<br>$2.18 \cdot 10^{-3}$<br>$2.09 \cdot 10^{-3}$<br>$2.00 \cdot 10^{-3}$<br>$1.88 \cdot 10^{-3}$<br>$1.63 \cdot 10^{-3}$<br>$1.48 \cdot 10^{-3}$<br>$1.37 \cdot 10^{-3}$	[19]

TABLE S6: EIS parameters obtained from the equivalent circuit for the 2-, 4-, 6- and 8-layer PG electrodes.

**Parameters**	**2 layers**	**4 layers**	**6 layers**	**8 layers**
$R_s/\Omega\ cm^2$	0.62	0.66	0.53	0.58
$R_{sl}/\Omega\ cm^2$	2.2	3.2	1.5	1.9
$C_{sl}/mF\ cm^{-2}$	$1.3\cdot10^{-4}$	$2.3\cdot10^{-4}$	$4.6\cdot10^{-4}$	$2.5\cdot10^{-4}$
$W_1/\ \Omega\ cm^2$	0.13	0.50	0.18	0.24
$R_{ct}/\ \Omega\ cm^2$	0.46	1.0	0.39	0.51
$C_{dl}/mF\ cm^{-2}$	$26\cdot10^{-3}$	$30\cdot10^{-3}$	$35\cdot10^{-3}$	$19\cdot10^{-3}$
$W_2/\Omega\ cm^2$	2.0	1.8	1.4	2.8
$C_p/mF\ cm^{-2}$	4.8	5.6	5.7	4.4

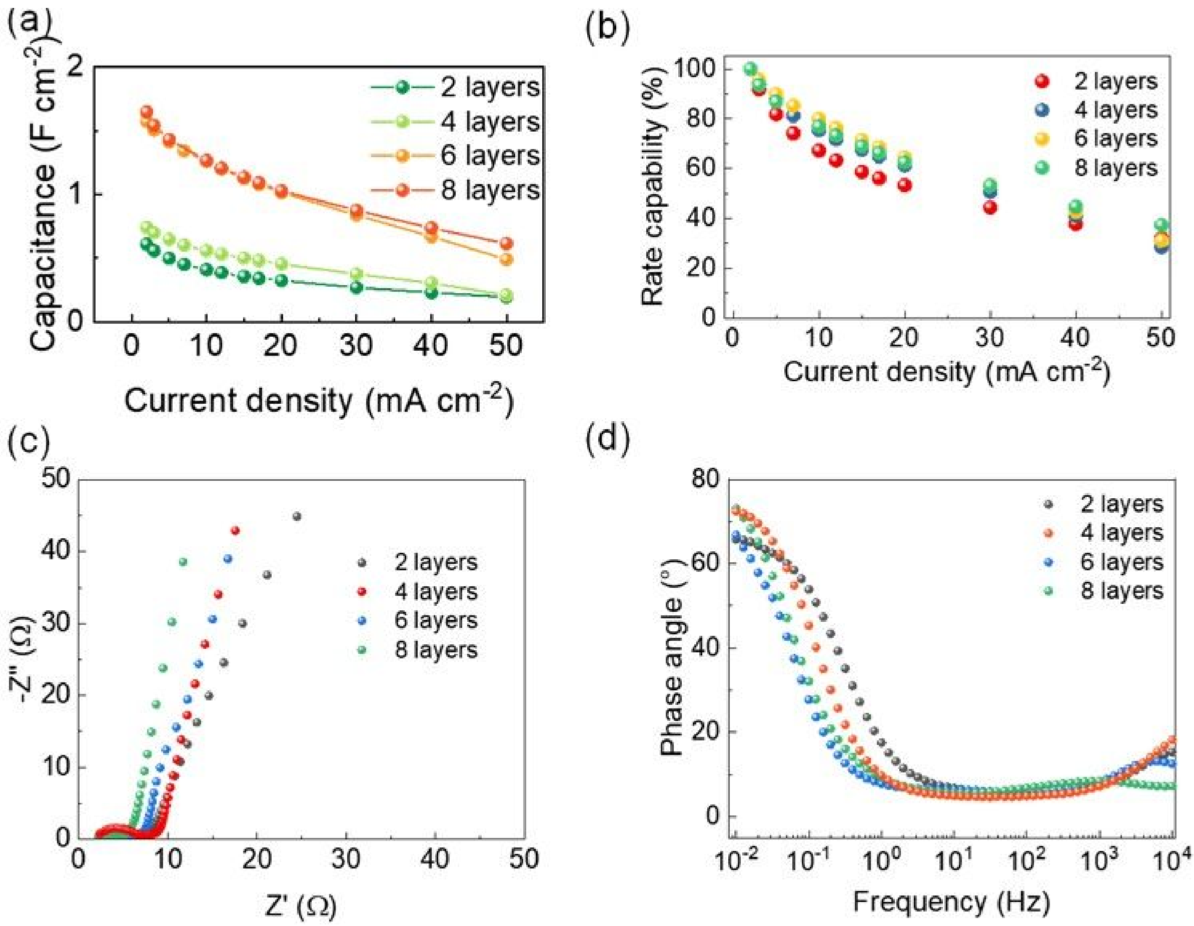

FIGURE S12: a,b) Areal capacitance (a) and rate capability (b) at increasing current densities for symmetric devices with different number of layers; c,d) Electrochemical impedance spectroscopy (c) and Bode plots (d) of symmetric devices with different number of layers.

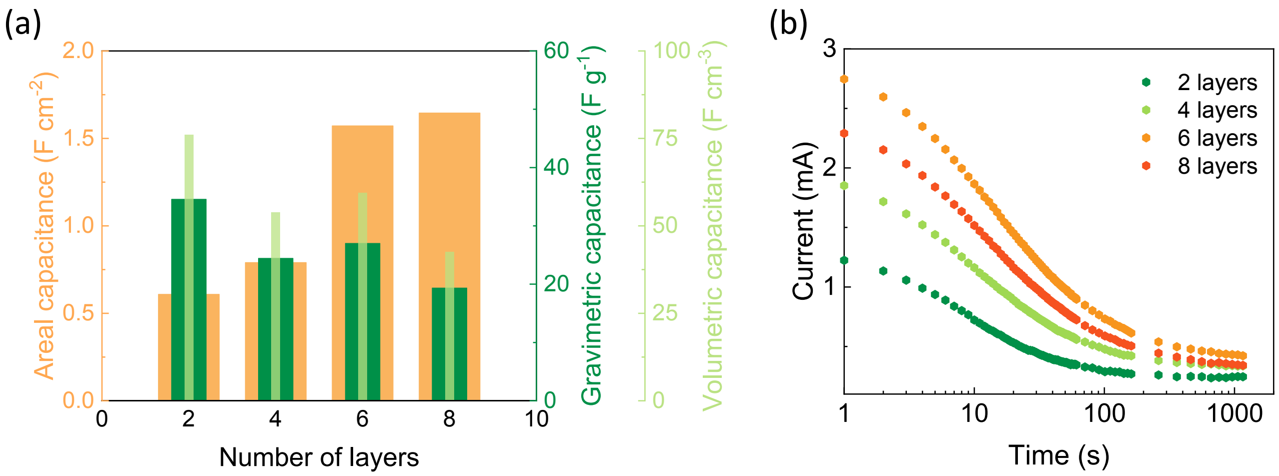

FIGURE S13: a) Comparison of areal, gravimetric and volumetric capacitance for symmetric devices (2 to 8 layers); b) Leakage current measured during the holding step of voltage holding tests for symmetric devices at different number of layers.

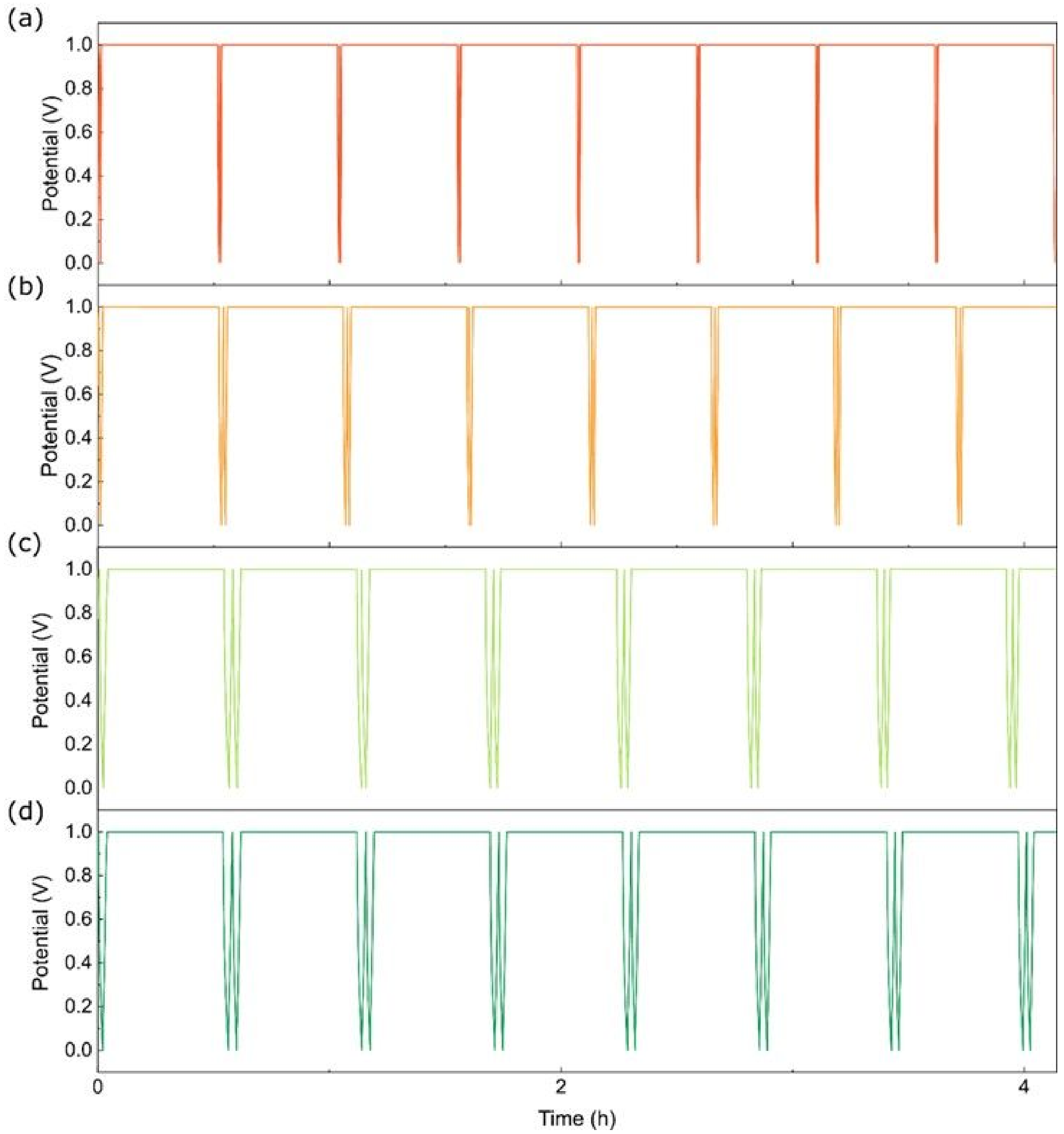

FIGURE S14: a-d) First 4h of the voltage holding tests on 2-layer (a), 4-layer (b), 6-layer (c) and 8-layer (d) symmetric devices.

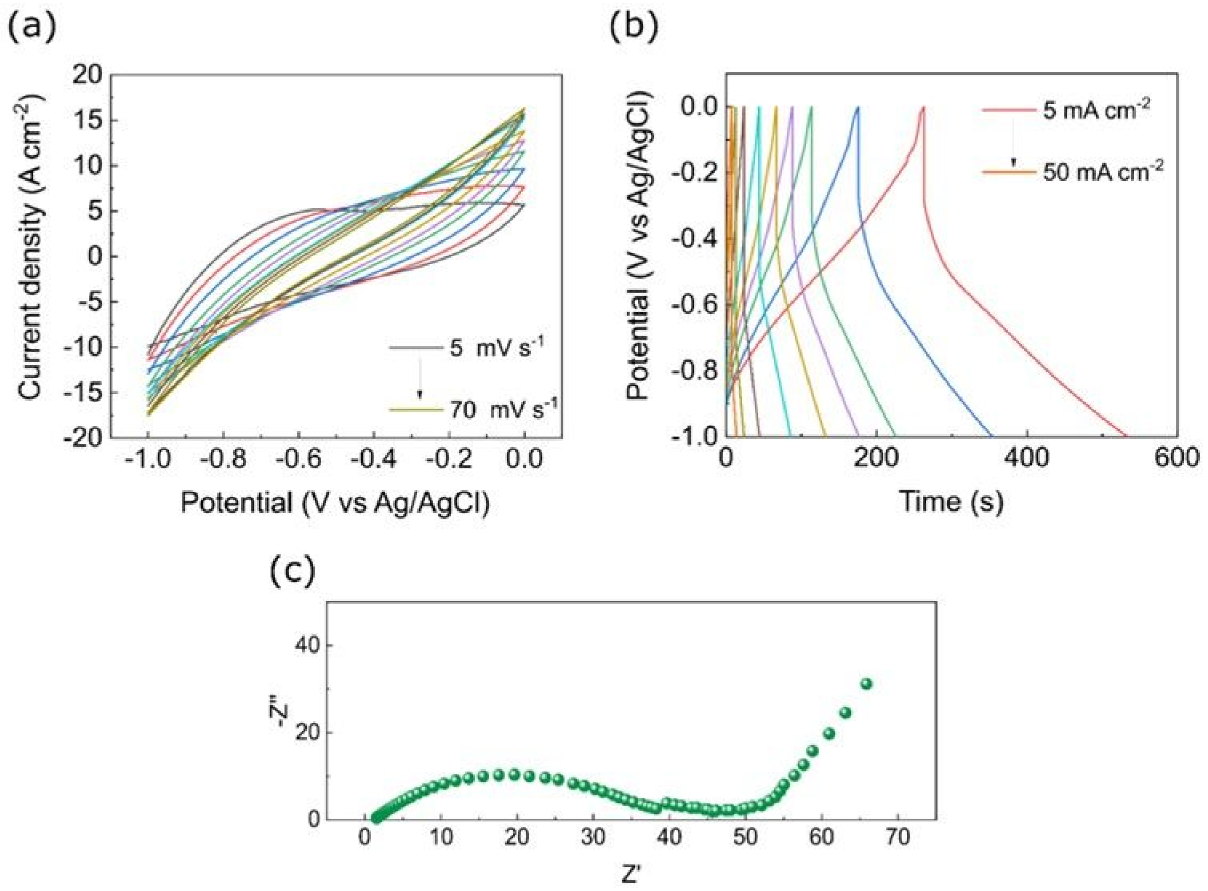

FIGURE S15: a) Cyclic voltammogram for the 4-layer compact structure exhibiting poor electrochemical performance; b) Galvanostatic charge-discharge curves for the 4-layer compact structure; c) EIS of the 4-layer compact structure showing high transport resistance

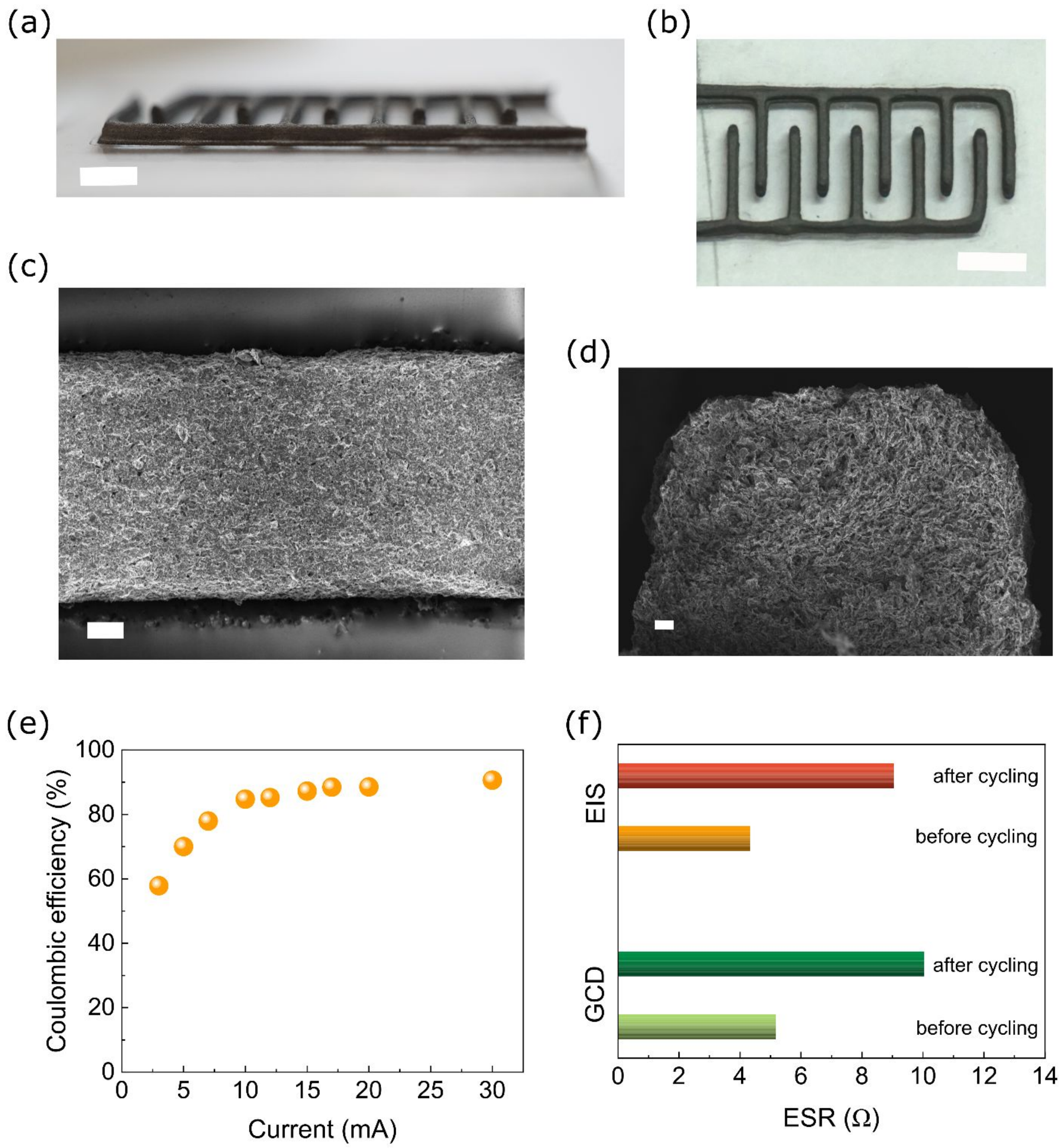

FIGURE S16: a,b) Pictures of the interdigitated electrodes printed on a microscope glass slide (scalebars 3 and 5 mm, respectively); c) SEM image of one finger of the interdigitated structure (scalebar 100 μm); d) Cross-section of one finger of the interdigitated structure (scalebar 30 μm); e) Coulombic efficiency of the interdigitated device at various charge-discharge currents; f) Equivalent Series Resistance (ESR) before and after cycling as calculated from charge-discharge and impedance spectroscopy.

In three-electrode tests, the specific capacitance was calculated from cyclic-voltammetry curves as:

$$C_s = \frac{\oint i \, \mathrm{d}V}{2 \, \nu \, x \, \Delta V} \# (\textbf{Equation S5})$$

where ΔV is the voltage window (1 V), ν the scan rate, x is either the area of the electrode (for areal capacitance), the volume of the electrode (volumetric capacitance) or the mass of the electrode (gravimetric capacitance) and the integral is extended to all the cycle considered.

The specific capacitance was also calculated from galvanostatic charge-discharge curve as:

$$C_s = \frac{i \, \Delta t}{x \, \Delta V} \# (\textbf{Equation S6})$$

where i is the discharge current, Δt the discharge time, ΔV the difference between the initial and final potential during discharge and x is define as above.

When testing symmetric devices, the specific capacitance was calculated from cyclic-voltammetry curves as:

$$C_{dev} = 4 \cdot \frac{\oint i \, \mathrm{d}V}{2 \, \nu \, \Delta V \, x} \# (\textbf{Equation S7})$$

where ΔV is the voltage window (1 V), ν the scan rate, x is either the area of the device (for areal capacitance), the volume of the two electrodes (volumetric capacitance) or the total mass of the two electrodes (gravimetric capacitance) and the integral is extended to all the cycle considered. The volume of the electrodes was calculated as:

$$V_{el} = \frac{m_{el}}{\rho} \# (\textbf{Equation S8})$$

where m_{el} is the measured mass of the electrodes and ρ is the density of the printed filaments (2.2 g cm^{-3}, measured *via* the buoyancy method)

The specific capacitance was also calculated from galvanostatic charge-discharge curve as:

$$C_{dev} = 4 \cdot \frac{i\,\Delta t}{x\,\Delta V} \#(\textbf{Equation S9})$$

where i is the discharge current, Δt the discharge time and ΔV the difference between the initial and final potential during discharge and x is defined as above.

The power and energy density of symmetric devices were calculated from galvanostatic charge-discharge as:

$$E_{dev} = \frac{1}{8} C_{dev} \Delta V^2 \#(\textbf{Equation S10})$$

$$P_{dev} = \frac{E_{dev}}{\Delta t} \#(\textbf{Equation S11})$$

where Δt is the discharge time.

TABLE S7: Areal capacitance, energy and power densities of the symmetric supercapacitor (2-8 layers) at 2 mA cm^{-2}.

**Number of layers**	**Capacitance ($F\ cm^{-2}$)**	**Energy density ($mWh\ cm^{-2}$)**	**Power density ($mW\ cm^{-2}$)**
2	0.61	0.02	0.98
4	0.74	0.02	0.95
6	1.57	0.05	0.97
8	1.65	0.05	0.98

TABLE S8: Gravimetric capacitance, energy and power densities of the symmetric supercapacitor (2-8 layers) at 2 mA cm^{-2}.

**Number of layers**	**Capacitance ($F g^{-1}$)**	**Energy density ($mWh g^{-1}$)**	**Power density ($mW g^{-1}$)**
2	34.58	1.14	55.46
4	21.66	0.68	27.79
6	27.01	0.88	16.64
8	19.36	0.64	11.49

TABLE S9: Volumetric capacitance, energy and power densities of the symmetric supercapacitor (2-8 layers) at 2 mA cm^{-2}.

**Number of layers**	**Capacitance ($F cm^{-3}$)**	**Energy density ($mWh cm^{-3}$)**	**Power density ($mW cm^{-3}$)**
2	76.08	2.52	122.02
4	47.65	1.49	61.13
6	59.41	1.94	36.61
8	42.58	1.41	25.27

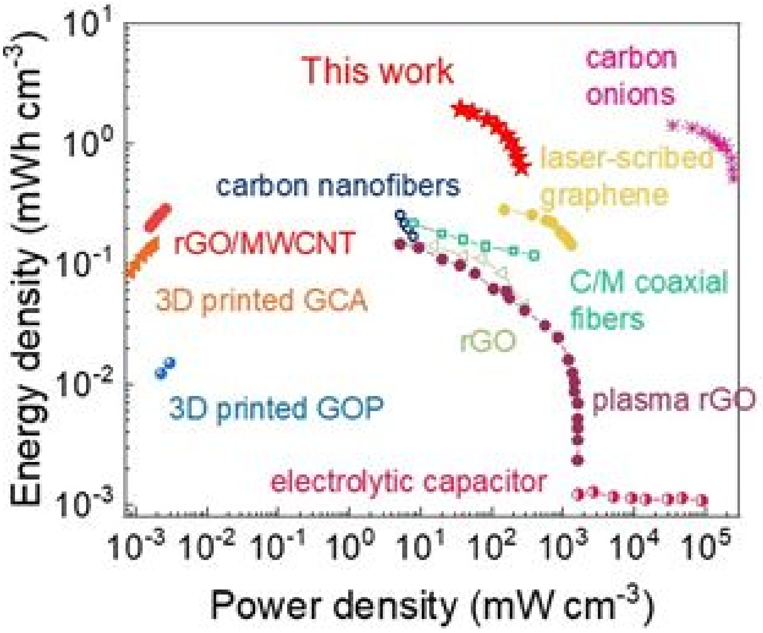

FIGURE S17: Volumetric energy and power densities of our 3D printed microsupercapacitor with previously reported microsupercapacitors.[11,34–37]

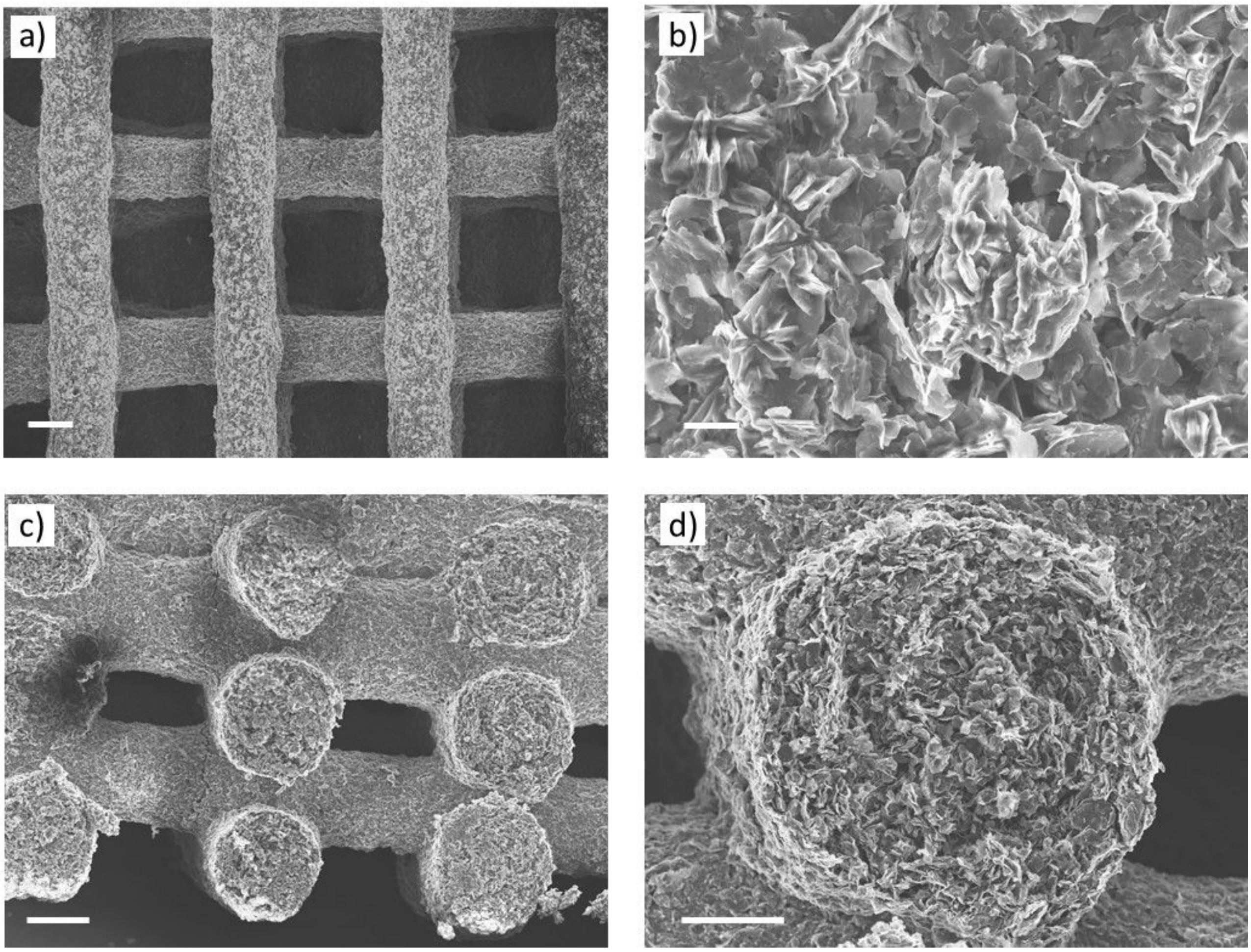

FIGURE S18: SEM images of the electrodes after 10000 cycles of charge and discharge (at 20 mA cm^{-1}): front view (a,b) and cross-section (c,d) view at different magnifications. Scalebars are 200 µm (a), 10 µm (b), 200 µm (c) and 100 µm (d).

REFERENCES:

(1) Casiraghi, C.; Pisana, S.; Novoselov, K. S.; Geim, A. K.; Ferrari, A. C. Raman Fingerprint of Charged Impurities in Graphene. *Appl. Phys. Lett.* **2007**, *91* (23), 233108. https://doi.org/10.1063/1.2818692.

(2) Ferrari, A. C.; Meyer, J. C.; Scardaci, V.; Casiraghi, C.; Lazzeri, M.; Mauri, F.; Piscanec, S.; Jiang, D.; Novoselov, K. S.; Roth, S.; Geim, A. K. Raman Spectrum of Graphene and Graphene Layers. *Phys. Rev. Lett.* **2006**, *97* (18), 187401. https://doi.org/10.1103/PhysRevLett.97.187401.

(3) Eckmann, A.; Felten, A.; Mishchenko, A.; Britnell, L.; Krupke, R.; Novoselov, K. S.; Casiraghi, C. Probing the Nature of Defects in Graphene by Raman Spectroscopy. *Nano Lett.* **2012**, *12* (8), 3925–3930. https://doi.org/10.1021/nl300901a.

(4) Gupta, A.; Chen, G.; Joshi, P.; Tadigadapa, S.; Eklund. Raman Scattering from High-Frequency Phonons in Supported n-Graphene Layer Films. *Nano Lett.* **2006**, *6* (12), 2667–2673. https://doi.org/10.1021/nl061420a.

(5) Yang, H.; Hernandez, Y.; Schlierf, A.; Felten, A.; Eckmann, A.; Johal, S.; Louette, P.; Pireaux, J.-J.; Feng, X.; Mullen, K.; Palermo, V.; Casiraghi, C. A Simple Method for Graphene Production Based on Exfoliation of Graphite in Water Using 1-Pyrenesulfonic Acid Sodium Salt. *Carbon N. Y.* **2013**, *53*, 357–365. https://doi.org/https://doi.org/10.1016/j.carbon.2012.11.022.

(6) Orangi, J.; Hamade, F.; Davis, V. A.; Beidaghi, M. 3D Printing of Additive-Free 2D Ti3C2Tx (MXene) Ink for Fabrication of Micro-Supercapacitors with Ultra-High Energy Densities. *ACS Nano* **2020**, *14* (1), 640–650. https://doi.org/10.1021/acsnano.9b07325.

(7) Yang, W.; Yang, J.; Byun, J. J.; Moissinac, F. P.; Xu, J.; Haigh, S. J.; Domingos, M.; Bissett, M. A.; Dryfe, R. A. W.; Barg, S. 3D Printing of Freestanding MXene Architectures for Current-Collector-Free Supercapacitors. *Adv. Mater.* **2019**, *31* (37), 1–8. https://doi.org/10.1002/adma.201902725.

(8) Jiang, Y.; Xu, Z.; Huang, T.; Liu, Y.; Guo, F.; Xi, J.; Gao, W.; Gao, C. Direct 3D Printing of Ultralight Graphene Oxide Aerogel Microlattices. *Adv. Funct. Mater.* **2018**, *28* (16), 1–8. https://doi.org/10.1002/adfm.201707024.

(9) Lacey, S. D.; Kirsch, D. J.; Li, Y.; Morgenstern, J. T.; Zarket, B. C.; Yao, Y.; Dai, J.; Garcia, L. Q.; Liu, B.; Gao, T.; Xu, S.; Raghavan, S. R.; Connell, J. W.; Lin, Y.; Hu, L. Extrusion-Based 3D Printing of Hierarchically Porous Advanced Battery Electrodes. *Adv. Mater.* **2018**, *30* (12), 1–9. https://doi.org/10.1002/adma.201705651.

(10) Li, W.; Li, Y.; Su, M.; An, B.; Liu, J.; Su, D.; Li, L.; Li, F.; Song, Y. Printing Assembly and Structural Regulation of Graphene towards Three-Dimensional Flexible Micro-Supercapacitors. *J. Mater. Chem. A* **2017**, *5* (31), 16281–16288. https://doi.org/10.1039/c7ta02041d.

(11) Zhou, H.; He, P.; Xie, P.; Cai, Z.; Fan, T.; Cheng, D.; Tang, X.; Zhang, D. Generalized 3D Printing of Graphene-Based Mixed-Dimensional Hybrid Aerogels. *ACS Nano* **2018**, *12* (4), 3502–3511. https://doi.org/10.1021/acsnano.8b00304.

(12) Yuk, H.; Lu, B.; Lin, S.; Qu, K.; Xu, J.; Luo, J.; Zhao, X. 3D Printing of Conducting Polymers.

Nat. Commun. **2020**, *11* (1), 4–11. https://doi.org/10.1038/s41467-020-15316-7.

(13) Daalkhaijav, U.; Yirmibesoglu, O. D.; Walker, S.; Mengüç, Y. Rheological Modification of Liquid Metal for Additive Manufacturing of Stretchable Electronics. *Adv. Mater. Technol.* **2018**, *3* (4), 1700351. https://doi.org/10.1002/admt.201700351.

(14) Cao, D.; Xing, Y.; Tantratian, K.; Wang, X.; Ma, Y.; Mukhopadhyay, A.; Cheng, Z.; Zhang, Q.; Jiao, Y.; Chen, L.; Zhu, H. 3D Printed High-Performance Lithium Metal Microbatteries Enabled by Nanocellulose. *Adv. Mater.* **2019**, *31* (14), 1–10. https://doi.org/10.1002/adma.201807313.

(15) Kyle, S.; Jessop, Z. M.; Al-Sabah, A.; Whitaker, I. S. 'Printability'' of Candidate Biomaterials for Extrusion Based 3D Printing: State-of-the-Art.' *Adv. Healthc. Mater.* **2017**, *6* (16), 1700264. https://doi.org/https://doi.org/10.1002/adhm.201700264.

(16) Chhabra, R. P.; Richardson, J. F. Flow in Pipes and in Conduits of Non-Circular Cross-Sections. In *Non-Newtonian Flow and Applied Rheology (Second Edition)*; Butterworth-Heinemann, 2008; pp 110–205. https://doi.org/10.1016/b978-075063770-1/50004-x.

(17) Tang, X.; Zhou, H.; Cai, Z.; Cheng, D.; He, P.; Xie, P.; Zhang, D.; Fan, T. Generalized 3D Printing of Graphene-Based Mixed-Dimensional Hybrid Aerogels. *ACS Nano* **2018**, *12* (4), 3502–3511. https://doi.org/10.1021/acsnano.8b00304.

(18) Zhang, C. (John); McKeon, L.; Kremer, M. P.; Park, S. H.; Ronan, O.; Seral-Ascaso, A.; Barwich, S.; Coileáin, C.; McEvoy, N.; Nerl, H. C.; Anasori, B.; Coleman, J. N.; Gogotsi, Y.; Nicolosi, V. Additive-Free MXene Inks and Direct Printing of Micro-Supercapacitors. *Nat. Commun.* **2019**, *10* (1), 1–9. https://doi.org/10.1038/s41467-019-09398-1.

(19) Sollami Delekta, S.; Smith, A. D.; Li, J.; Östling, M. Inkjet Printed Highly Transparent and Flexible Graphene Micro-Supercapacitors. *Nanoscale* **2017**, *9* (21), 6998–7005. https://doi.org/10.1039/C7NR02204B.

(20) Liu, Z.; Wu, Z.-S.; Yang, S.; Dong, R.; Feng, X.; Müllen, K. Ultraflexible In-Plane Micro-Supercapacitors by Direct Printing of Solution-Processable Electrochemically Exfoliated Graphene. *Adv. Mater.* **2016**, *28* (11), 2217–2222. https://doi.org/https://doi.org/10.1002/adma.201505304.

(21) Wu, Z.; Parvez, K.; Feng, X.; Müllen, K. Graphene-Based in-Plane Micro-Supercapacitors with High Power and Energy Densities. *Nat. Commun.* **2013**, *4* (1), 2487. https://doi.org/10.1038/ncomms3487.

(22) Gao, W.; Singh, N.; Song, L.; Liu, Z.; Reddy, A. L. M.; Ci, L.; Vajtai, R.; Zhang, Q.; Wei, B.; Ajayan, P. M. Direct Laser Writing of Micro-Supercapacitors on Hydrated Graphite Oxide Films. *Nat. Nanotechnol.* **2011**, *6* (8), 496–500. https://doi.org/10.1038/nnano.2011.110.

(23) Wu, Z.-S.; Tan, Y.-Z.; Zheng, S.; Wang, S.; Parvez, K.; Qin, J.; Shi, X.; Sun, C.; Bao, X.; Feng, X.; Müllen, K. Bottom-Up Fabrication of Sulfur-Doped Graphene Films Derived from Sulfur-Annulated Nanographene for Ultrahigh Volumetric Capacitance Micro-Supercapacitors. *J. Am. Chem. Soc.* **2017**, *139* (12), 4506–4512. https://doi.org/10.1021/jacs.7b00805.

(24) Wang, S.; Wu, Z.-S.; Zheng, S.; Zhou, F.; Sun, C.; Cheng, H.-M.; Bao, X. Scalable Fabrication of Photochemically Reduced Graphene-Based Monolithic Micro-Supercapacitors with Superior Energy and Power Densities. *ACS Nano* **2017**, *11* (4), 4283–4291. https://doi.org/10.1021/acsnano.7b01390.

(25) Shao, Y.; Li, J.; Li, Y.; Wang, H.; Zhang, Q.; Kaner, R. B. Flexible Quasi-Solid-State Planar Micro-Supercapacitor Based on Cellular Graphene Films. *Mater. Horiz.* **2017**, *4* (6), 1145–1150. https://doi.org/10.1039/C7MH00441A.

(26) Lin, J.; Zhang, C.; Yan, Z.; Zhu, Y.; Peng, Z.; Hauge, R. H.; Natelson, D.; Tour, J. M. 3-Dimensional Graphene Carbon Nanotube Carpet-Based Microsupercapacitors with High Electrochemical Performance. *Nano Lett.* **2013**, *13* (1), 72–78. https://doi.org/10.1021/nl3034976.

(27) Beidaghi, M.; Wang, C. Micro-Supercapacitors Based on Interdigital Electrodes of Reduced Graphene Oxide and Carbon Nanotube Composites with Ultrahigh Power Handling Performance. *Adv. Funct. Mater.* **2012**, *22* (21), 4501–4510. https://doi.org/https://doi.org/10.1002/adfm.201201292.

(28) Huang, P.; Lethien, C.; Pinaud, S.; Brousse, K.; Laloo, R.; Turq, V.; Respaud, M.; Demortière, A.; Daffos, B.; Taberna, P. L.; Chaudret, B.; Gogotsi, Y.; Simon, P. On-Chip and Freestanding Elastic Carbon Films for Micro-Supercapacitors. *Science (80-.).* **2016**, *351* (6274), 691–695. https://doi.org/10.1126/science.aad3345.

(29) Xu, Y.; Lin, Z.; Huang, X.; Liu, Y.; Huang, Y.; Duan, X. Flexible Solid-State Supercapacitors Based on Three-Dimensional Graphene Hydrogel Films. *ACS Nano* **2013**, *7* (5), 4042–4049. https://doi.org/10.1021/nn4000836.

(30) Xiong, Z.; Liao, C.; Han, W.; Wang, X. Mechanically Tough Large-Area Hierarchical Porous Graphene Films for High-Performance Flexible Supercapacitor Applications. *Adv. Mater.* **2015**, *27* (30), 4469–4475. https://doi.org/https://doi.org/10.1002/adma.201501983.

(31) Shao, Y.; El-Kady, M. F.; Lin, C.-W.; Zhu, G.; Marsh, K. L.; Hwang, J. Y.; Zhang, Q.; Li, Y.; Wang, H.; Kaner, R. B. 3D Freeze-Casting of Cellular Graphene Films for Ultrahigh-Power-Density Supercapacitors. *Adv. Mater.* **2016**, *28* (31), 6719–6726. https://doi.org/https://doi.org/10.1002/adma.201506157.

(32) Le, V. T.; Kim, H.; Ghosh, A.; Kim, J.; Chang, J.; Vu, Q. A.; Pham, D. T.; Lee, J.-H.; Kim, S.-W.; Lee, Y. H. Coaxial Fiber Supercapacitor Using All-Carbon Material Electrodes. *ACS Nano* **2013**, *7* (7), 5940–5947. https://doi.org/10.1021/nn4016345.

(33) Zhu, C.; Liu, T.; Qian, F.; Han, T. Y. J.; Duoss, E. B.; Kuntz, J. D.; Spadaccini, C. M.; Worsley, M. A.; Li, Y. Supercapacitors Based on Three-Dimensional Hierarchical Graphene Aerogels with Periodic Macropores. *Nano Lett.* **2016**, *16* (6), 3448–3456. https://doi.org/10.1021/acs.nanolett.5b04965.

(34) Acerce, M.; Voiry, D.; Chhowalla, M. Metallic 1T Phase MoS2nanosheets as Supercapacitor Electrode Materials. *Nat. Nanotechnol.* **2015**, *10* (4), 313–318. https://doi.org/10.1038/nnano.2015.40.

(35) Xiao, X.; Li, T.; Yang, P.; Gao, Y.; Jin, H.; Ni, W.; Zhan, W.; Zhang, X.; Cao, Y.; Zhong, J.; Gong, L.; Yen, W.-C.; Mai, W.; Chen, J.; Huo, K.; Chueh, Y.-L.; Wang, Z. L.; Zhou, J. Fiber-Based All-Solid-State Flexible Supercapacitors for Self-Powered Systems. *ACS Nano* **2012**, *6* (10), 9200–9206. https://doi.org/10.1021/nn303530k.

(36) Li, L.; Secor, E. B.; Chen, K.-S.; Zhu, J.; Liu, X.; Gao, T. Z.; Seo, J.-W. T.; Zhao, Y.; Hersam, M. C. High-Performance Solid-State Supercapacitors and Microsupercapacitors Derived from Printable Graphene Inks. *Adv. Energy Mater.* **2016**, *6* (20), 1600909. https://doi.org/https://doi.org/10.1002/aenm.201600909.

(37) Pech, D.; Brunet, M.; Durou, H.; Huang, P.; Mochalin, V.; Gogotsi, Y.; Taberna, P.-L.; Simon, P. Ultrahigh-Power Micrometre-Sized Supercapacitors Based on Onion-Like Carbon. *Nat. Nanotechnol.* **2010**, *5* (9), 651–654. https://doi.org/10.1038/nnano.2010.162.
